# A likely geological record of deep tremor and slow slip events from a subducted continental broken formation

**DOI:** 10.1038/s41598-022-08489-2

**Published:** 2022-03-16

**Authors:** Francesco Giuntoli, Giulio Viola

**Affiliations:** Department of Biological, Geological and Environmental Sciences, Università degli Studi di Bologna, Bologna, Italy

**Keywords:** Geology, Structural geology, Petrology

## Abstract

Fluids in subduction zones play a key role in controlling seismic activity, drastically affecting the rheology of rocks, triggering mineral reactions, and lowering the effective stress. Fluctuating pore pressure is one important parameter for the switch between brittle and ductile deformation, thus impacting seismogenesis. Episodic tremor and slow slip events (ETS) have been proposed as a common feature of the geophysical signature of subduction zones. Their geological record, however, remains scanty. Only the detailed and further characterization of exhumed fossil geological settings can help fill this knowledge gap. Here we propose that fluctuating pore pressure linked to metamorphic dehydration reactions steered cyclic and ETS-related brittle and ductile deformation of continental crustal rocks in the subduction channel of the Apennines. Dilational shear veins and ductile mylonitic shear zones formed broadly coevally at minimum 1 GPa and 350 °C, corresponding to ~ 30–40 km depth in the subduction zone. We identify carpholite in Ca-poor metasediments as an important carrier of H_2_O to depths > 40 km in cold subduction zones. Our results suggest that the described (micro)structures and mineralogical changes can be ascribed to deep ETS and provide a useful reference for the interpretation of similar tectonic settings worldwide.

## Introduction

Subduction zones effectively control the volatile cycle between shallow crust and mantle. Fluids are liberated in subduction zones at shallow depth primarily due to compaction and porosity decrease and at greater depth to dehydration via mineralogical reactions (e.g.^1,2^) of the subducting slab. Fluid release has major implications for volcanic activity, ore-deposit formation, metamorphic reactions and the rheological behaviour of deforming rocks and related seismicity.

Episodic tremor and slow slip events (ETS) are constituent elements of the geophysical record of subduction zones. Tremors are a persistent low-amplitude seismic signal associated with slow slip, that is, a slip larger than the average plate motion that is detected geodetically, lasting from days to years^3–5^. Their seismic signature has been related to deforming rock volumes characterised by high V_p_/V_s_ ratios, where pore pressure may transiently reach and even exceed lithostatic values^6,7^. ETS are located in the immediate surroundings of megathrust earthquakes and likely play a role as stress meters and in the stress transfer to the megathrust fault^8^, although a spatial separation between ETS and megathrust earthquakes is also reported from some warm subduction zones^9^.

 Despite the growing number of seismological studies, the evidence in the geological record of ETS remains little constrained and poorly understood and thus is the basis for a lively debated^10,11^. Behr and Bürgmann^11^ and Kirkpatrick et al.^10^ suggest a series of potential geological structures related to ETS, reminding us that thus far there is no universally accepted deformation structure that can be considered positively diagnostic for ETS in the exhumed geological record of subduction zones. Subduction-related heterogeneities such as “block-in-matrix” type fabrics, or tightly juxtaposed lithologies with different rheology, widespread evidence of broadly coeval alternating brittle and ductile deformation, and of cyclically elevated pore pressure and low differential stress might all be direct geological records of ETS. The geological record that we can access, however, is the end result of a long evolution occurring at different depths, temperatures or time throughout the subduction history and its interpretation is always challenging. Hitherto, the geological structures that are potentially ascribable to ETS are documented from accretionary wedge domains^12–14^ or subducted oceanic crust^15–17^, whereas they are scanty in subducted continental crust^18,19^. Further geological work is, therefore, necessary to better characterize the record of ETS in fossil geological settings^10,11^ and use it to refine the understanding of active megathrusts.

Here we present original data from the fossil subduction interface of the Northern Apennines (Italy), specifically from subducted continental metasediments deformed at blueschist facies conditions. These metasediments are composed of heterometric, rheologically strong blocks embedded in a weaker matrix containing dilational shear veins and ductile mylonitic shear zones formed broadly coevally. Thermodynamic modelling constrains the formation of both veins and mylonites to high pressure conditions (at least 1 GPa and 350 °C). We conclude that these structures formed due to cyclic brittle and ductile deformation steered by fluctuating pore pressure, transiently reaching near-lithostatic values. These structures might represent a geological record of deep ETS, as defined by^11^, in continental metasediments.

## Results

### A likely geological record of deep ETS in continental metasediments

The Apennines formed due to the convergence and successive collision of the European and African plates from the Late Eocene (e.g.^20^, Supplementary Fig. 1). The internal Northern Apennines are characterized by unmetamorphosed continental and oceanic units stacked with their metamorphic counterparts. The metamorphosed oceanic units derive from the Ligure-Piemontese oceanic basin, whereas the continental units originate from the former Tethyan passive distal continental margin of the stretched Adria plate (e.g.^21,22^). Initial subduction of oceanic crust was followed by subduction of this distal continental margin, as attested to by metamorphic high pressure-low temperature conditions between 0.8–1.6 GPa and 300–500 °C (summary of pressure and temperature (P–T) conditions in^23^). In particular, carpholite and lawsonite in the metamorphic parageneses from metamorphosed continental and oceanic units suggest relatively cold geothermal gradients of c. 8–10 °C/km during subduction (e.g.^22,24^).

**Figure 1:**
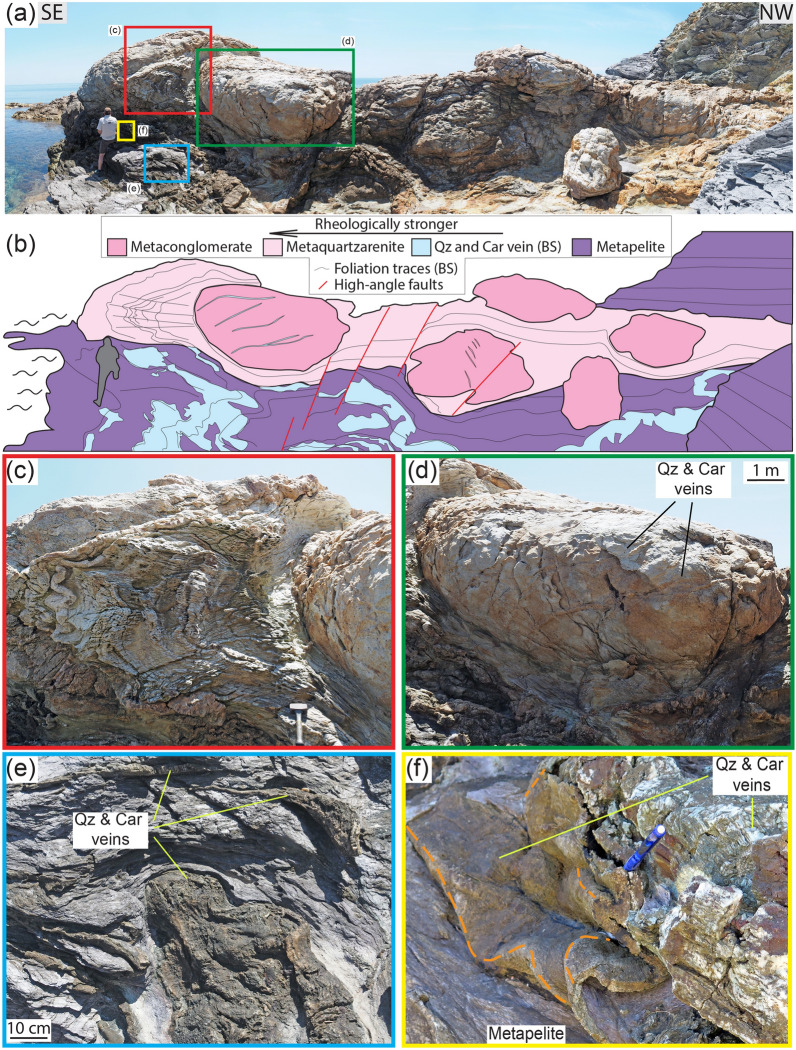
Outcrop (**a**) and interpretative sketch (**b**) of the Verrucano broken formation, with boudins of competent lithotypes (metaconglomerate, metaquartzarenite) embedded within weak metapelite. Quartz and carpholite veins occur in both metaconglomerate and metapelite. BS = blueschist facies conditions, see text for details. Giglio Island (Tuscany, Italy). (**c**) Detail of the contact between metaconglomerate boudin (right) and intensely and disharmonically folded metaquartzarenite. Folding style is likely due to the stark competence contrast causing local kinematic flow perturbations (see section “*Block in matrix”* behaviour for further details). (**d**) Detail of metaconglomerate boudin with quartz and carpholite veins arranged in an en-echelon array. (**e**) Metapelite with folded dilational hydroshear veins composed of quartz and carpholite fibres. (**f**) Detail of non-cylindrical folding of dilational hydroshear vein. Dashed orange lines indicate curvilinear fold hinges; the pen is oriented subparallel to the stretching lineation. Mineral abbreviation in figures from^43^. Figure (**b**) created with Adobe Illustrator CS6 (https://www.adobe.com/products/illustrator.html).

**Figure 2:**
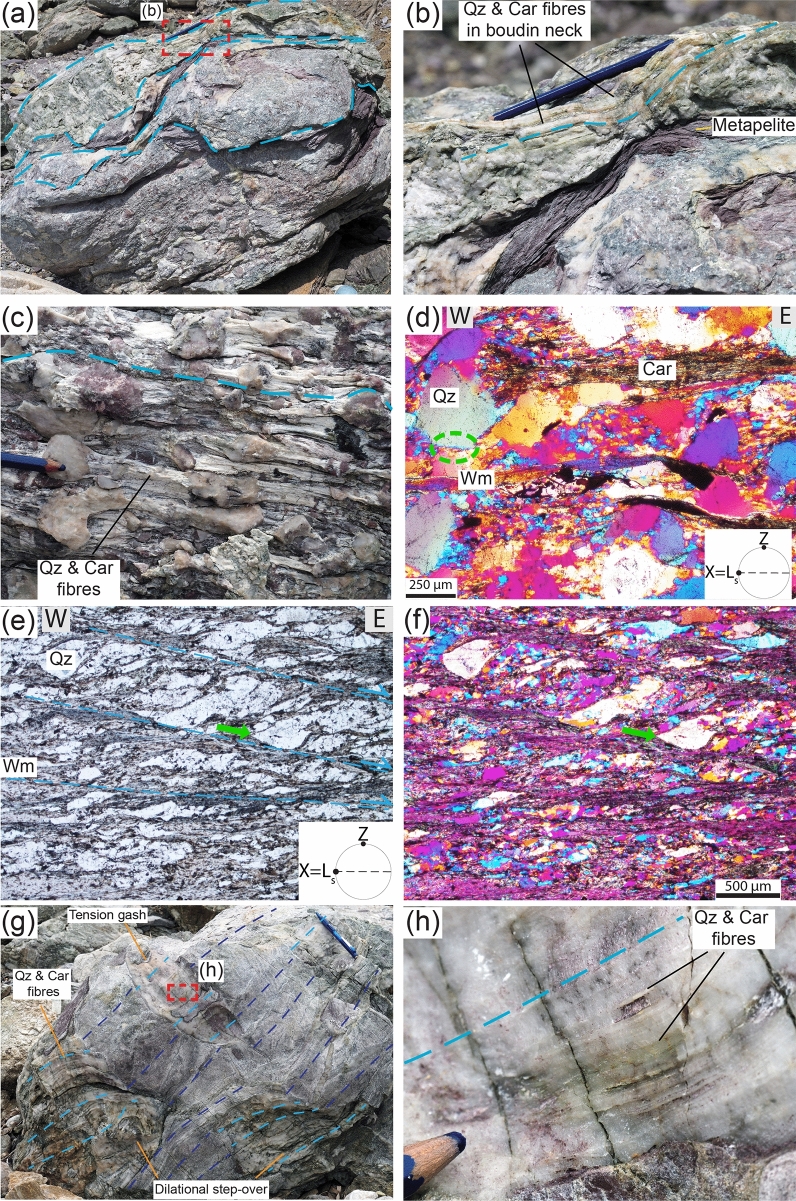
Brittle and ductile deformation features affecting the studied lithotypes. (**a**) Metaconglomerate boudins within metapelite layers; the contact between the two lithotypes are indicated by the dashed light blue lines. (**b**) Quartz and carpholite fibres grow iso-oriented in boudin necks (dashed light blue lines). (**c**) Metaconglomerate with rounded quartz clasts, from pink to white in colour, displaying quartz and carpholite fibres growing in the asymmetric strain shadows of the clasts. (**d**) Thin section photo with detrital quartz clasts wrapped by the blueschist facies foliation producing asymmetric pressure shadows marked by carpholite and white mica. The dashed green ellipsis indicates two compenetrated quartz clasts. Crossed-polarized light with gypsum plate inserted. (**e**,**f**) Thin section photos of metaquartzarenite with detrital quartz clasts sheared by blueschist facies top-to-the-E extensional crenulation cleavage (light blue dashed lines) marked by white mica. Detrital quartz clasts are overgrown epitaxially in the asymmetric pressure shadows (green arrow). Plane-, crossed-polarized light with gypsum plate inserted, respectively. (**g**) Metaquartzarenite block containing tension gashes and dilational step-overs sealed by quartz and carpholite fibres growing parallelly to the blueschist facies foliation. Dashed light blue lines indicate the orientation of the fibres, dashed dark blue lines the trace of foliation. (**h**) Detail of tension gash with quartz (white) and carpholite (greenish-yellowish) fibres, some centimetres long.

The metamorphosed continental units in the study area are composed of a several 100-m-thick Middle-Low Triassic metasedimentary clastic sequence comprising alternating metaconglomerate, metaquartzarenite and metapelite layers and is regionally referred to as Verrucano Formation^25^ (Supplementary Fig. 1d). The Verrucano Formation (traditionally called “Verrucano") is indeed of regional importance as it crops out over an area of ~ 12,000 km^2^ in the Northern Apennines. In the study area, the Verrucano is intensely deformed, with mechanically competent metaconglomerate and metaquartzarenite layers stretched and boudinaged and enveloped by less competent metapelite (Figs. 1a,b and 2a,b; GPS coordinates in Supplementary Table 1). As a whole, it forms a so-called “broken formation”, defined as a “disrupted rock unit with a block-in-matrix fabric, which contains no exotic blocks but only native components”^26,27^ (Fig. 1c,d). The observed structures crop out over an area of ~ 1 km^2^.

 Diffuse ductile deformation is therein characterized by shear zones at all scales, with a mylonitic blueschist facies foliation defined by aligned carpholite, phengitic muscovite (Si content > 3 apfu) and quartz wrapping around clastic quartz grains and producing asymmetric pressure shadows composed of quartz, carpholite and muscovite (Fig. 2c–f and Supplementary Fig. 2; details in Supplementary). The metaconglomerate and metaquartzarenite may contain quartz clasts displaying evidence of compenetration at sites of high normal stress and asymmetric pressure shadows occurring at sites of low normal stress (i.e., perpendicular and parallel to the foliation, respectively). In between two compenetrated grains, phyllosilicates may locally occur and in the pressure shadows quartz is finer grained and overgrows epitactically the clasts (Fig. 2d–f). These microstructures evidence deformation mainly by dissolution–precipitation creep (e.g.^28^).

**Figure 3:**
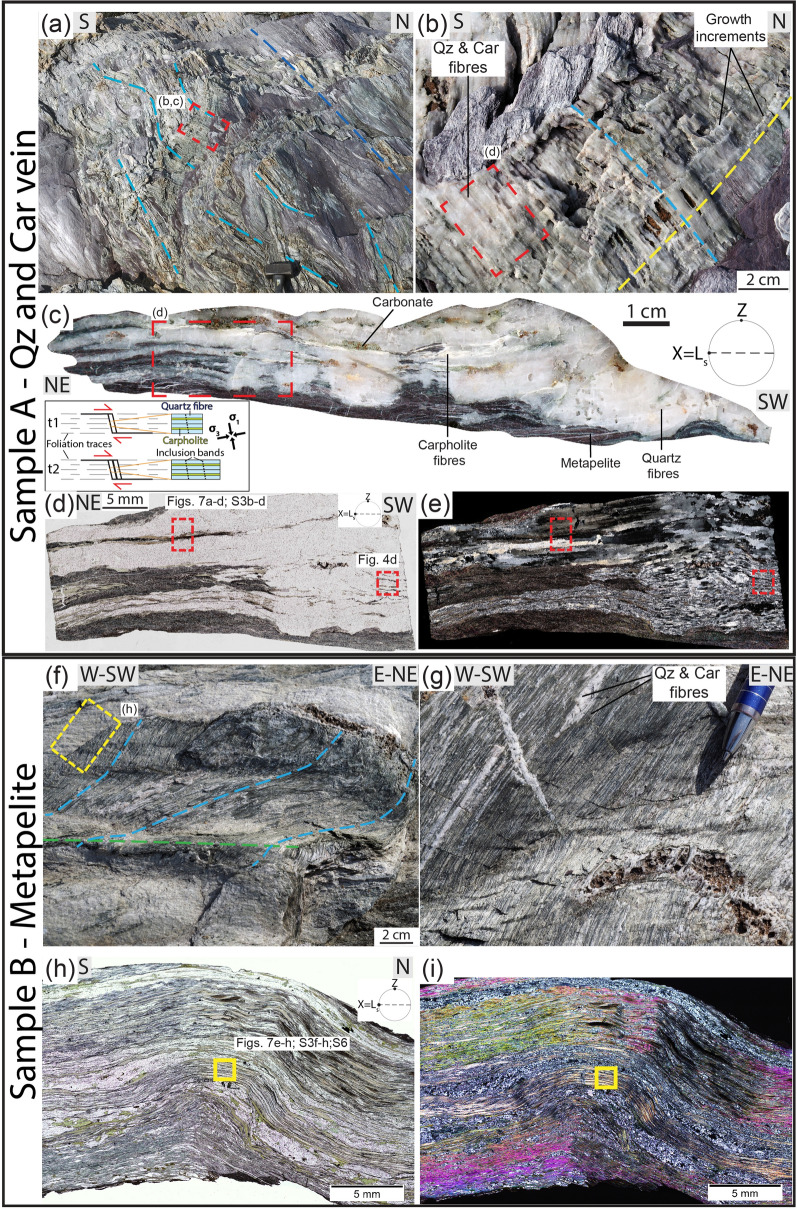
Meso and microstructural features of dilational hydroshear vein and embedding metapelite. (**a**) Metapelite with quartz and carpholite dilational hydroshear veins, some metres in length. View parallel to foliation plane; dashed light blue and blue lines indicate fibres and stretching lineation, respectively. (**b**) Detail of dilational hydroshear vein with individual quartz and carpholite fibres up to 10 cm long. Carbonate is locally intergrown with the previous minerals. Note crack-seal growth increments marked by fractures oriented parallel to the vein boundaries and highlighted by the dashed yellow line. (**c**) Polished slab of (**b**) cut parallel to both stretching lineation and quartz and carpholite fibres (X = L_s_) and perpendicular to the foliation (Z parallel to the pole of the mylonitic foliation). Incipient lateral segmentation and boudinage of the quartz band is evident along the bottom of the hydroshear vein. The sketch illustrates the formation mechanism of dilational hydroshear veins, with inclusion bands marking growth increments oriented perpendicular to the long dimension of the crystals and vein boundaries; assumed stress trajectories (σ_1_ and σ_3_) are shown (based on^31^). (**d**,**e**) Thin section optical scans of (**c**) with quartz bands composed of fibres some centimetres in length. Some fibres show undulose extinction. Mica-rich bands appear dark due to micron-sized inclusions of hematite and graphite. Carpholite fibres vary from colourless to brownish due to incipient retrogression. Plane‐polarized light and crossed-polarized light, respectively. (**f**) Stretching lineation in metapelite defined by quartz and carpholite fibres on a foliation plane (dashed light blue lines) deformed by upright fold (axial plane trace: dashed light green line); view parallel to foliation plane. (**g**) Detail of quartz (white) and carpholite (green) fibres intergrown with muscovite (silvery). Weathered carbonate (brown) is located inside a vein. (**h**,**i**) Thin section optical scans of (**f**) displaying a folded foliation defined by quartz-rich- and white mica and carpholite-rich bands. Chlorite, with green absorption colours, locally replaces carpholite (further details in Supplementary Figs. 3e–h and 6). Plane‐polarized light and crossed-polarized light, respectively.

 Localized brittle deformation is also common and is expressed by tension gashes, dilational step-overs and dilational shear veins occurring in the three lithotypes. Dilational step-overs and tension gashes occur mainly in the stronger lithotypes oriented at high angle to the foliation (~ 70–80°; Fig. 2g,h), whereas dilational shear veins are subparallel to the foliation and are more common in metapelite (Fig. 3a–e). In the metaconglomerate and metaquartzarenite, dilational shear veins may be up to 1 m long, a few decimetres thick and can form up to ~ 10% of the entire volume of the rock. In the metapelite, dilational shear veins can be several metres long and up to a few decimetres thick and locally represent 50% of the rock volume. In all lithotypes vein geometry is from straight to winding (Figs. 1, 2g,h and 3a–e).

**Figure 4:**
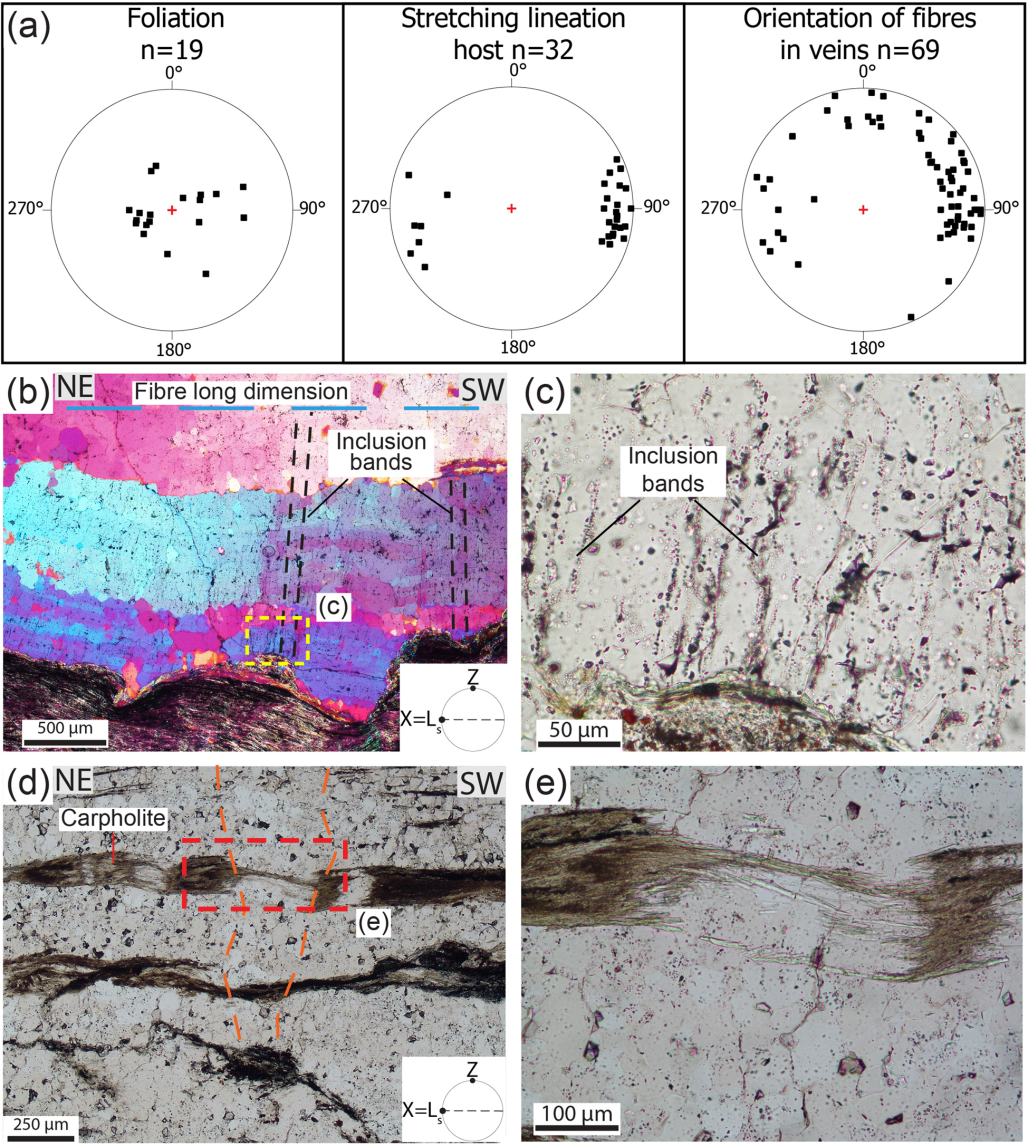
Stereographic projections of selected planar (poles to foliation planes) and linear fabric elements and microstructural details of dilational hydroshear vein in sample A. (**a**) Equal area, lower hemisphere stereographic projections. Poles to foliation planes, stretching lineation in metapelite, metaquartzarenite and metaconglomerates and orientation of fibres in veins. (**b**) The quartz band in dilational hydroshear vein is rich in inclusion bands (black dashed lines) oriented perpendicular to long dimension of the fibres (dashed light blue line) and vein boundaries, which indicates vein growth by incremental crack-seal (see main text). Crossed-polarized light with gypsum plate inserted. (**c**) Detail of the inclusion bands and the white mica rich in graphite and hematite inclusions (bottom part of the photo). Plane-polarized light. (**d**) Stretched and partly boudinaged carpholite fibres in dilational hydroshear vein. The boudin necks are infilled by quartz and finer grained carpholite. The boundaries of a boudin neck are marked by the dashed orange line. (**e**) Detail of a boudin neck highlighting incremental crack-sealing stages. Plane-polarized light.

The veins are composed of quartz and carpholite fibres, some decimetres long, and minor carbonate (Figs. 2g,h and 3a,b). The fibres are iso-oriented, and parallel and perpendicular to the vein boundaries in the dilational shear veins and tension gashes, respectively. They are also parallel to the host-rock stretching lineation, which is defined by phengitic muscovite, quartz and carpholite grains. Both vein fibres and stretching lineation plunge gently to the E-NE, with only a few scattered vein fibres plunging to the N; the mylonitic foliation dips gently to the E (Fig. 4a). Dilational shear veins exhibit crack-and-seal textures. At the outcrop, fibres are locally cut across by thin sets of parallel fractures oriented perpendicularly to the fibre long dimension (i.e. perpendicular to the stretching direction of the fibres) and perpendicular to the foliation, marking successive growth increments (Figs. 2h and 3b).

In thin section, quartz fibres appear as up to a few centimetres long and display undulose extinction (Figs. 3e and 4b). They are monocrystalline, as visible in the optical scans of the thin sections with the gypsum plate inserted, and have high aspect ratios (up to 30; Supplementary Fig. 3a). They are locally recrystallized, leading to a significant grain size reduction down to some tens of microns and low aspect ratios for the new grains. Quartz fibres contain abundant fluid inclusion bands with both liquid and vapor phases^29^ oriented perpendicular to the long dimension of the grains (i.e. perpendicular to the foliation; Fig. 4b,c and Supplementary Fig. 4a–d). Spacing between the inclusion bands is in the order of a few tens of microns, although some variability exists between adjacent bands. Similar microfractures are also present in the carpholite fibres (see next sections), although along those fractures fluid inclusions bands are not observed. This type of microstructure has been interpreted by^12,30^ as resulting from incremental crack-and-seal growth of the dilational shear veins (see also sketch of Fig. 3c). In the text below, these veins will be referred to as dilational hydroshear veins, as defined by^31^. Additionally, phengitic muscovite layers occur between quartz and carpholite fibres, suggesting opening of the dilational hydroshear veins at high angle to the foliation along the low-shear strength foliation planes that are locally exploited as slip surfaces, according to the model of^12,32^ (Fig. 3c–e). Summarizing, these microstructures reflect the incremental epitaxial growth of the fibres by repeated brittle failure and sealing of the fracture coupled to slip along the phengitic muscovite-rich bands during overall ductile deformation of the host rock (see sketch in Fig. 3c).

**Figure 5:**
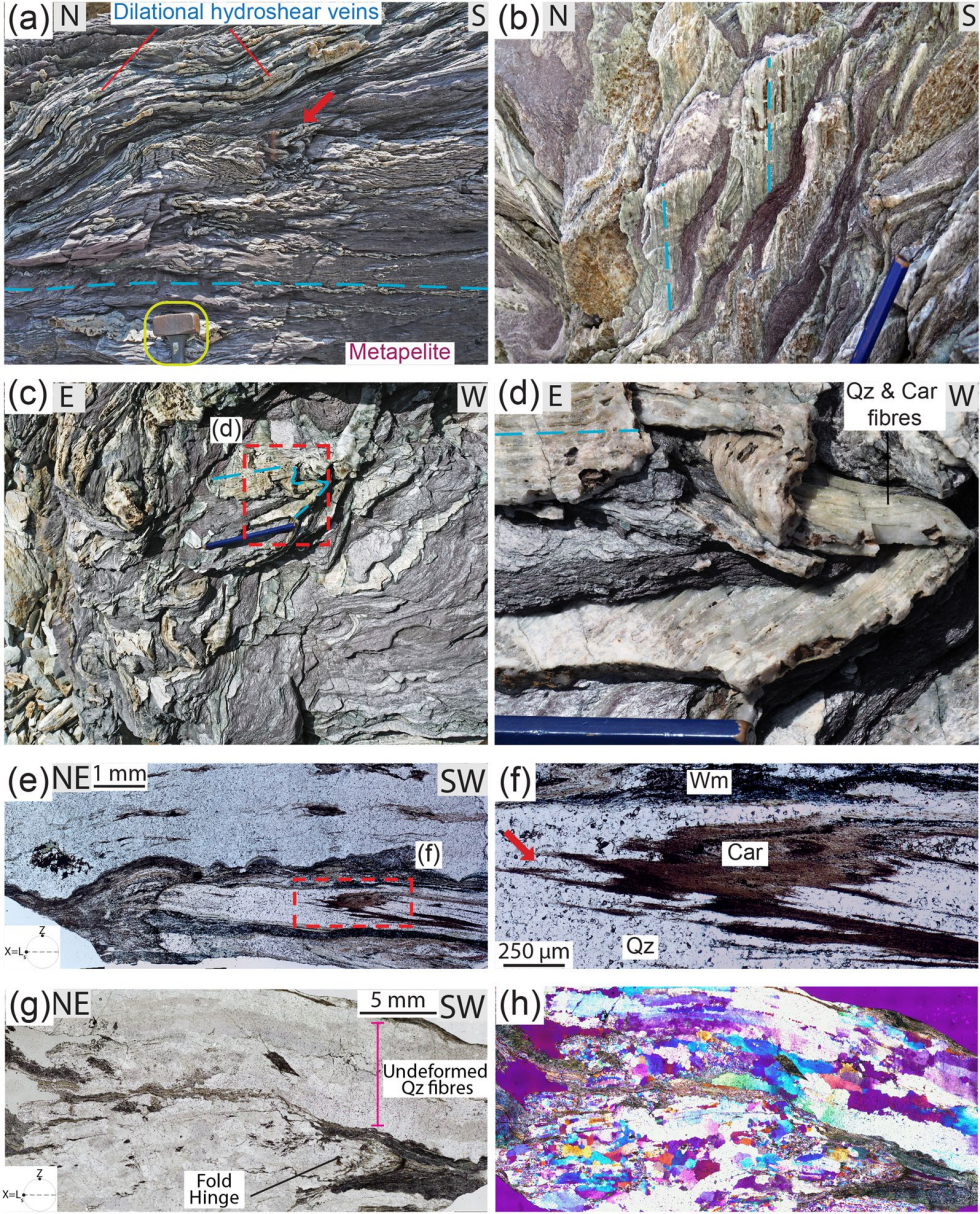
Metapelite with blueschist facies foliation and folded quartz and carpholite dilational hydroshear veins. (**a**) View perpendicular to the foliation. The light blue dashed line indicates the trace of the blueschist facies foliation, the red arrow highlights a fold hinge; yellow circle: hammer for scale. (**b**) View parallel to foliation planes with iso-oriented quartz and carpholite fibres in hydroshear veins (light blue dashed lines). (**c**,**d**) Detail of non-cylindrical folding of dilational hydroshear vein with quartz and carpholite fibres up to 10 cm long. (**e**,**f**) Thin section photos of dilational hydroshear vein with fold defined by white mica-rich bands. The red arrow in (**f**) points at carpholite grains in the hinge zone oriented parallel to the axial plane of the fold. Plane-polarized light. (**g**,**h**) The thin section is composed of an isoclinal fold surrounded by phengitic muscovite bands (bottom) and almost undeformed quartz fibres some centimetres long with undulose extinction (top part). Plane- and crossed-polarized light with gypsum plate inserted, respectively.

**Figure 6:**
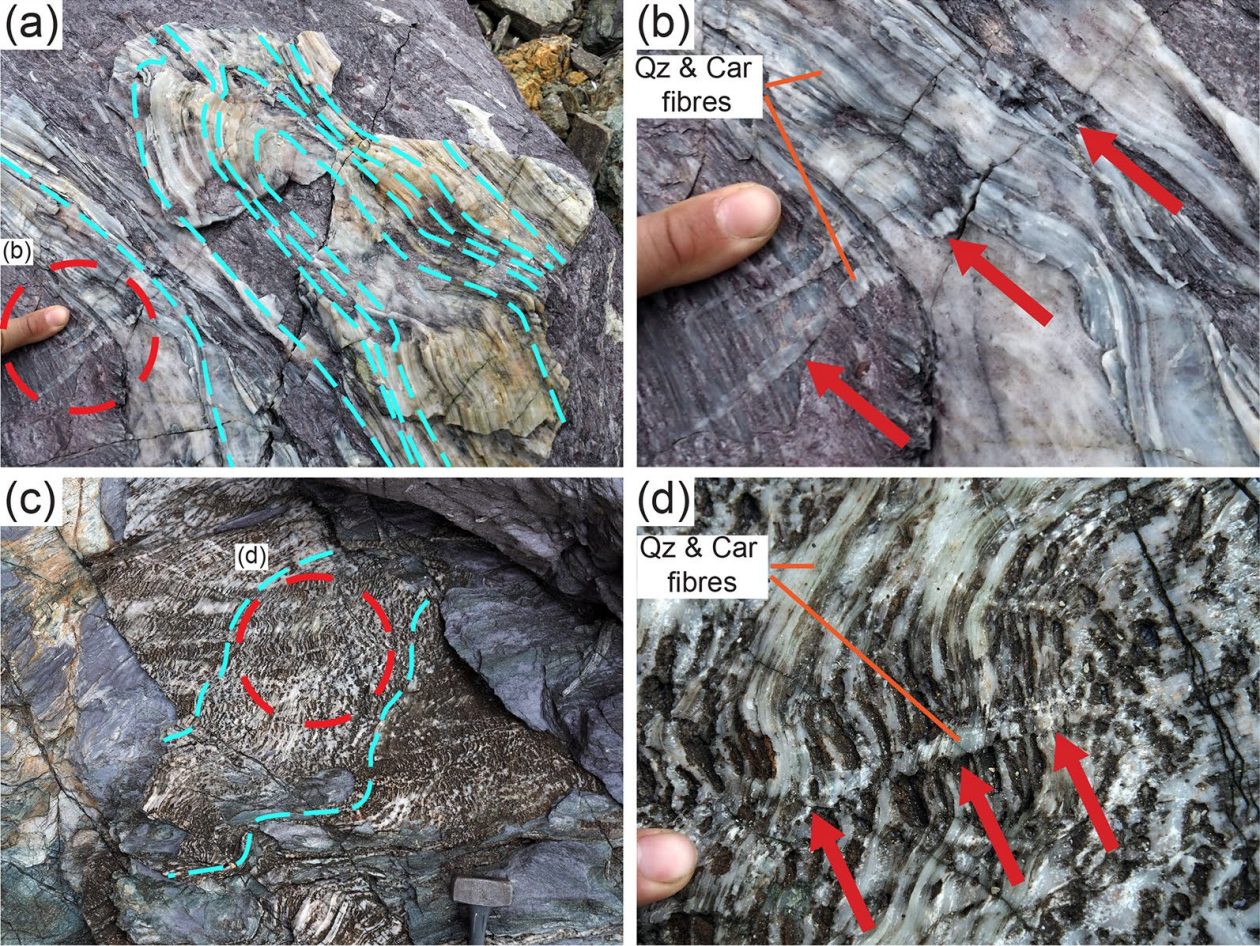
Non-cylindrical folding of dilational hydroshear veins cut by a younger generation of quartz and carpholite veins (red arrows) in metaconglomerate (**a**,**b**) and metapelite (**c**,**d**). The dashed light blue lines indicate the orientation of quartz and carpholite fibres in folded dilational hydroshear veins.

Locally, quartz bands display incipient boudinage, with muscovite in the boudin necks (Figs. 3c and 4b and Supplementary Fig. 4a–d). Carpholite fibres are also boudinaged and finer-grained carpholite and quartz occur in the boudin necks, suggesting that boudinage formed coevally with the documented incremental cracking and sealing (Fig. 4d,e and Supplementary Fig. 4e–h). Some veins are locally intensely folded by non-cylindrical folds, both at the meso- and microscale (Figs. 1f and 5). Carpholite is stable within fold hinges and is oriented parallel to the axial plane, supporting the conclusion that carpholite was stable at the time of folding (Fig. 5e,f). Additionally, folding is limited to some bands, leaving surrounding veins undeformed and suggesting that several episodes of dilational hydroshear vein formation occurred (Fig. 5g,h). Locally, folded dilational hydroshear veins are cut by a younger generation of quartz and carpholite veins (Fig. 6). Based on the presented meso- and microstructural data, several episodes of vein formation are to be postulated and cyclic brittle and ductile deformation are likely to have alternated at similar P–T conditions (see below).

### Dilational hydroshear veins forming at 30–40 km of depth

**Figure 7:**
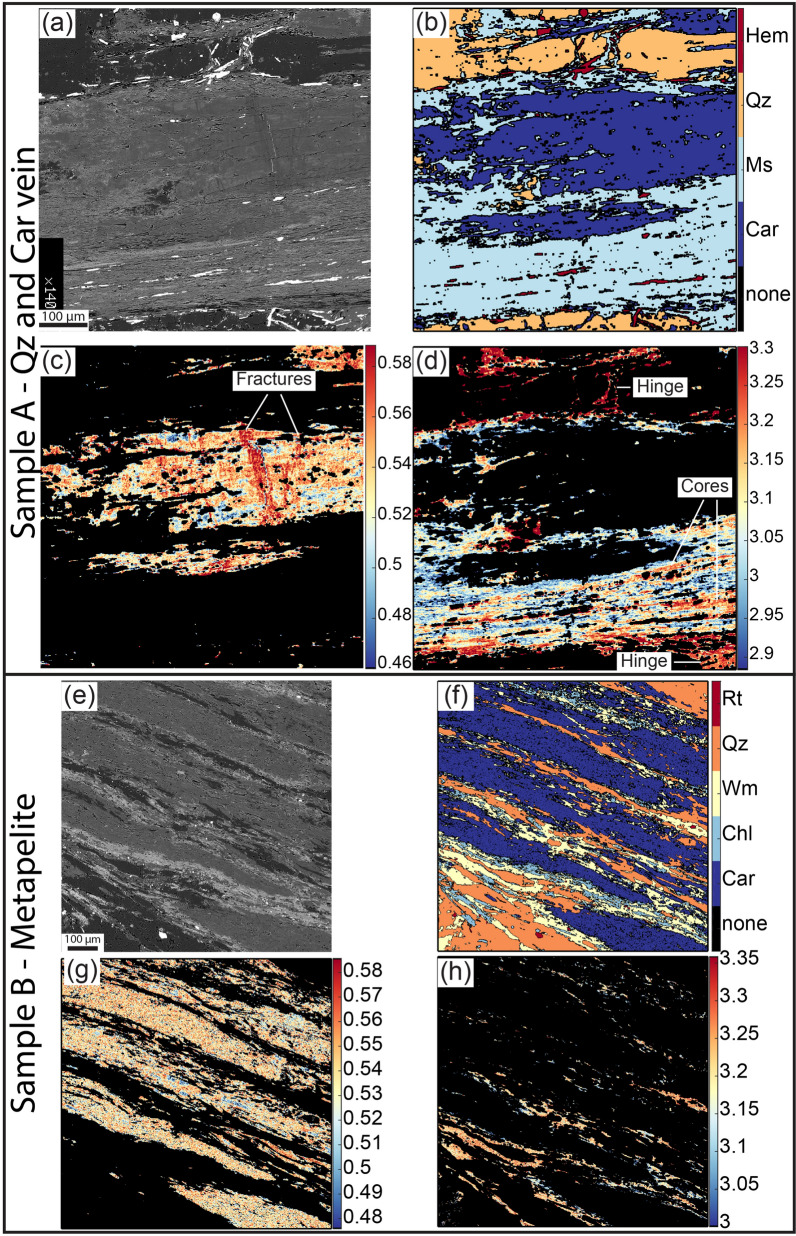
Chemical data from mineral phases infilling the dilational hydroshear veins and defining the mylonitic foliation. (**a**–**d**) Details of Fig. 3d,e. (**a**) BSE image showing fractures perpendicular to the carpholite fibre sealed by darker carpholite (compare with **b** and **c**). (**b**) X-ray compositional map, colour coded for the different mineral phases. (**c**) Standardised X-ray map of the X_Mg_ (X_Mg_ = Mg/(Fe^2+^ + Mg) in carpholite. Note the fractures oriented perpendicularly to the lengthening of the grain sealed by a second generation of carpholite with higher X_Mg_. (**d**) Muscovite Si apfu map highlights two muscovite generations: the first with higher values located in the core of the grains and in fold hinges and the second with lower values at grain boundaries. (**e**–**h**) Details of Fig. 3h,i. (**e**,**f**) BSE image and X-ray compositional map displaying a foliation marked by carpholite, muscovite and quartz, with chlorite growing at grain boundaries and marking retrogression. (**g**) Carpholite X_Mg_ map. (**h**) Muscovite Si apfu map. The lower values are located in proximity of chlorite grains.

The presence of syn-deformational carpholite in the dilational hydroshear veins suggests that fracturing and veining occurred at blueschist facies conditions, i.e. at high pressure and low temperature within the subduction channel (e.g.^24,33^). Quantitative compositional X-ray mapping reveals the presence of microcracks in the carpholite grains oriented perpendicular to the long dimension of the grains (i.e., perpendicular to the stretching direction) that are sealed by a second generation of carpholite with higher X_Mg_ content (X_Mg_ = Mg/(Fe^2+^ + Mg); Fig. 7a–c and Supplementary Table 2). This is consistent with the quartz microstructure constraining vein growth by incremental crack-seal, with repeated fracturing and sealing of the dilational hydroshear veins by mineral precipitation, as proposed by^12,30^ (Figs. 3b,c and 4b–e and Supplementary Fig. 4).

**Figure 8:**
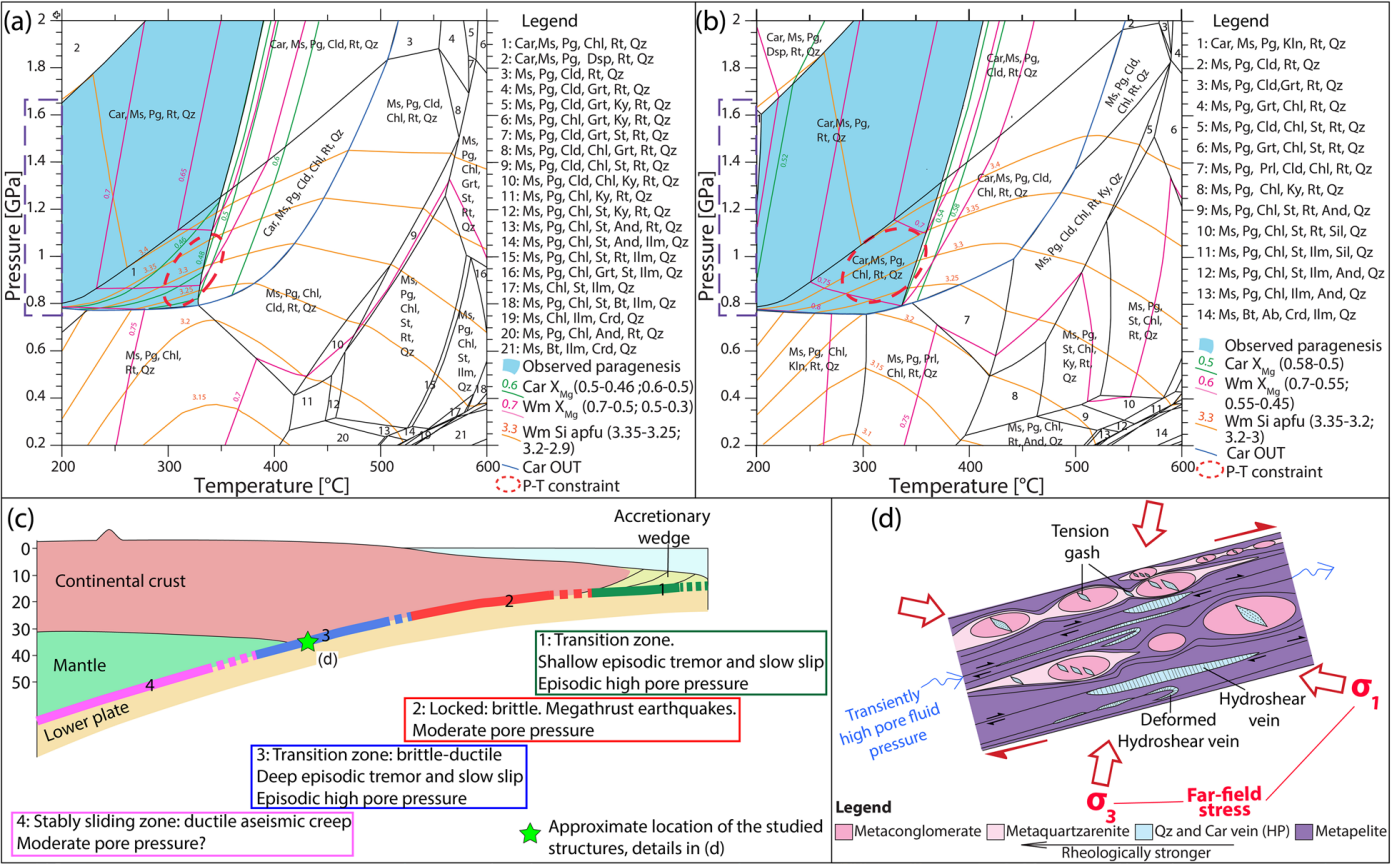
Thermodynamic modeling and conceptual sketch of the proposed geological record of ETS. (**a**,**b**) Equilibrium phase diagrams for quartz and carpholite dilational hydroshear vein (Sample A) and metapelite (Sample B), respectively. Red dashed ellipses indicate the P‐T conditions of the metamorphic stage best fitting the observed paragenesis and the computed isopleths. Pressure values corresponding to the depth range of deep ETS is indicated by the dashed violet rectangle (as constrained by^11^) by the dashed rectangle along the P axis. (**c**) Sketch of the main slip modes occurring along a subduction zone with location of the studied ETS (based on^6^). (**d**) Sketch of the proposed geological record of deep ETS (based on^36^; created with Adobe Illustrator CS6 https://www.adobe.com/products/illustrator.html).

Thermodynamic modelling was carried out using an X-ray mapping approach that allowed us to extract the local bulk composition of the studied microstructures^34,35^ (see “Methods”; Fig. 7 and Supplementary Fig. 3 and Table 3). Results constrain P–T conditions of at least 1 GPa and 300–350 °C for the formation of both the high-pressure veins (Sample A, Fig. 3a–e) and the mylonitic foliation in the metapelite (Sample B, Fig. 3f–i), similarly to what previously reported for this geological unit^29^ (Fig. 8a,b; details available in Supplementary).

## Discussion

The Verrucano bears evidence of brittle dilational hydroshearing and ductile shearing alternating cyclically during subduction at temperatures typical of the brittle-ductile transition zone (350–450 °C in^36,37^). Importantly, this zone can occur at greater depths than usual in cold subduction zones, where, as in the case of the Northern Apennines, a low geothermal gradient of 8–10 °C/km was present (data from this study and from^22,23,29^). Both veins and foliation formed at pressure conditions typical of c. 30–40 km depth. This depth corresponds to the lower limit of the seismogenic zone of some subduction megathrusts, where deep ETS are generally reported (e.g.^5,11,38^; Fig. 8c). Examples of ETS occurring in subduction settings with comparable geothermal gradients as in the Northern Apennines include the seismologically active modern subduction zones of New Zealand, Costa Rica, Alaska and SW Japan as well as fossil and exhumed subduction settings such as the Cyclades in Greece and the Italian Western Alps (Fig. 1 of^10^ and Fig. 3 of^11^ and references therein). Additionally, deep ETS are also reported from seismologically active modern subduction zones involving continental crust. This is the case of the southern Central Range of Taiwan, characterised by warmer geothermal gradients compared to the Northern Apennines. In this geological context, deep ETS occur between 15 and 45 km and are linked to metamorphic dehydration reactions happening within the subducting continental crust and producing high pore pressure and failure along low dipping thrust faults^39,40^.

Dilational hydroshear veins in subducted metasediments are thus interpreted as possible evidence of ETS and suggest the presence of pore pressure transiently exceeding the least principal compressive stress, roughly equivalent to near-lithostatic values in subduction settings^12,32,36^. Hence, we propose that the studied brittle structures likely correspond to the geological record of tremors documented by the seismologic data, while ductile deformation relates to slow slip in the geodetic data, as suggested by^7,12,15,32,41^. Moreover, our estimated P–T range corresponds to conditions typical for dehydration reactions in Ca-poor metasediments. Between 0.5 GPa–200 °C and 1.9 GPa–550 °C, the main hydrous minerals in Ca-poor metasediments are kaolinite (containing 14 weight percent (wt%) of H_2_O bounded in the crystal structure), pyrophyllite (5 wt%), chlorite (10–14 wt%), carpholite (10–11.5 wt%), chloritoid (7 wt%), muscovite (4.3 wt%) and paragonite (4.6 wt%; Fig. 8a,b). Above 0.8 GPa, carpholite can form from chlorite, kaolinite and pyrophyllite destabilization through the following reactions^42^ (mineral abbreviation from^43^):

1 $$ {\text{Ms}} + {\text{Chl}} + {\text{Qz}} + {\text{H}}_{{2}} {\text{O}} = {\text{Cel}} + {\text{Car}} $$

2 $$ {\text{Chl}} + {\text{Kln}} = {\text{Car}} + {\text{Qz}} + {\text{H}}_{{2}} {\text{O}} $$

3 $$ {\text{Chl}} + {\text{Prl}} + {\text{H}}_{{2}} {\text{O}} = {\text{Car}} + {\text{Qz}} $$

Between 350 and 550 °C, carpholite progressively destabilizes to form chloritoid:

4 $$ {\text{Car}} = {\text{Cld}} + {\text{Qz}} + {\text{H}}_{{2}} {\text{O}} $$

A direct correlation exists between the carpholite modal amount (volume percent-vol%) and the H_2_O content of the mineral phases (wt%) above 0.8 GPa (Supplementary Fig. 5). In our samples, carpholite decreases from 42 and 63 vol% (sample A and B, respectively) to 0 vol% between 1 GPa–300 °C and 1.8 GPa–500/550 °C (Supplementary Fig. 5c,h). This decrease matches the content of H_2_O in solids, from 7–8 wt% for sample A and B, respectively, to 4.6 wt% for both samples (Supplementary Fig. 5b,g). For our P–T conditions, the decrease in carpholite is balanced by chloritoid increase (Supplementary Fig. 5e,l). However, the net aqueous fluid release from the sample is positive, due to a lower amount of H_2_O per formula unit of chloritoid compared to carpholite.

Carpholite-rich veins can, therefore, act as a syn-deformation trap for H_2_O released from chlorite-out reactions, because a significant amount of H_2_O can be stored in the carpholite structural formula. Successively, this aqueous fluid can be released deeper down in the subduction channel, at a temperature higher than 350 °C, as the carpholite-out reaction is temperature dependent (Fig. 8a,b). Therefore, especially in cold subduction zones, carpholite can be stable down to ~ 60 km. Our estimates of 2.4–3.4 wt% of H_2_O release due to the carpholite-out reaction are maximum amounts. We intentionally selected samples that represent the higher spectrum of the carpholite vol% visible in the field, and we modelled dilational hydroshear veins and metapelite characterised by carpholite-rich bands (Supplementary Table 3). However, ~ 0.5–1 wt% of H_2_O release can be regarded as representative for the Verrucano broken formation, assuming a 5–15 vol% of carpholite as average value.

We thus propose carpholite to be one important H_2_O carrier in poor-Ca metasediments. This possibility has been overlooked^2^ or considered minor^44^, likely due to the restricted carpholite stability field, which is limited to cold subduction zones and poor-Ca metasediments, and to the ready destabilization of carpholite as temperature increases at the beginning of exhumation due to the relaxation of depressed isotherms^24^. We suggest carpholite to have destabilized in the studied fossil subduction channel starting only a few km deeper than the investigated samples, thus affecting the local rheological behaviour of the subduction interface. Most likely, also the dehydration of nearby oceanic units contributed to the fluid budget of the studied subduction interface, as above 350 °C the lawsonite-out dehydration reaction occurs (e.g.^2,45–48^). These data suggest that the described metamorphic dehydration reactions provide batches of aqueous fluid whenever a subducting volume of rocks crosses a dehydration reaction, leading to pore pressure fluctuations and brittle-ductile cyclicity, as proposed by^14,45,49,50^ and as also suggested for the Northern Apennines^51^.

The studied subduction interface is characterized by a broken formation, with more competent blocks in a weaker matrix. This is a scale-invariant feature, which is readily recognised from the macro- and meso- to the micro scale (Figs. 1, 2a,b and 3c–e). In metaconglomerate and metarenite, detrital quartz grains behave as rigid objects in a weaker ductile matrix composed of newly crystallised quartz, carpholite and muscovite grains growing in the strain shadows (Fig. 2c–f). Carpholite is stronger than quartz and is locally fractured and boudinaged, with quartz filling the boudin necks (Fig. 4d,e and Supplementary Fig. 4e–h). Quartz layers are more competent than the adjacent muscovite bands and display incipient boudinage (Figs. 3c and 4b,c). Such a “block in matrix” behaviour is similar to what described in^52^ for shallower geological settings. We cannot exclude that a “block in matrix” structure could have also been acquired at shallow structural levels at the onset of subduction^27^ and/or during the exhumation path “en route” to surface. However, the described ubiquitous foliation and dilational hydroshear veins and, more importantly, the presence of synkinematic carpholite and phengitic muscovite in boudins, boudin necks and matrix (Figs. 2a,b and 3c–e) constrain formation of these structures to the peak P–T recorded by these rocks. A later retrograde greenschist facies overprint is locally observed at the micro- and meso scale, where both blueschist facies foliation and veins are deformed by upright folds and S-C-C′ planes associated with chlorite (Supplementary Figs. 6 and 7; details available in Supplementary).

Episodic jamming of more competent blocks can occur during deformation, forming a transient load-bearing, more competent network of competent blocks and minor deformation in the surrounding weak matrix^53^. Jamming can cause fracturing of the blocks and surrounding matrix and distributed ductile deformation in the matrix^53^. Nevertheless, the peculiarity of the studied field site is that structures ascribable to both brittle and ductile deformation are found in all lithotypes, irrespective of their competence (Fig. 8d), comparable to what observed in metasediments deformed at similar temperatures but at much shallower depths^12^. This style of deformation is different from what reported by^15,41^, where brittle deformation is reported as only occurring in the more competent blocks, and ductile deformation is mostly limited to the surrounding weaker matrix.

In the Verrucano, metapelite and phyllosilicate-rich layers acted as seals that maintained overpressure and allowed for preferential fluid flow parallel to the mesoscopic planar anisotropy defined by the foliation and lithological boundaries^50^. Such conditions are highly favourable for the opening of dilational hydroshear veins, once critical overpressure conditions are met^32^. Successively, the stark competence contrast between blocks and matrix in the Verrucano likely induced local kinematic flow perturbations, inducing the non-cylindrical folding of both dilational hydroshear veins and foliation (Figs. 1c and 5). Finally, folded dilational hydroshear veins are cut by a younger generation of quartz and carpholite veins, attesting to the cyclic character of both brittle and ductile deformation (Fig. 6).

Concluding, we propose that the studied geological structures may represent the fossil record of deep ETS, which would have led to cyclic brittle and ductile deformation within the subduction channel at > 30 km depth. Pore pressure is proposed as the main trigger of cyclicity, repeatedly and transiently reaching near-lithostatic values due to metamorphic dehydration reactions occurring in both oceanic and continental rocks. We identify the carpholite-out reaction as one such reaction for metasediments poor in Ca, and carpholite as one of the carriers of H_2_O down to 30–60 km depth in cold subduction zones. Phyllosilicate-rich rocks can act as relatively low-permeability barriers that maintain overpressure and allow for fluid flow to preferentially occur parallel to the foliation and lithological boundaries. Our results suggest reconsidering the role of quartz-carpholite veins forming coevally with metamorphic foliation as a possible record of deep ETS in similar geological settings of other convergent orogens, including the Alps^54^, Tukey^55^, Crete^56^, Oman^57^, the Svalbard^24^ and New Caledonia^58^.

## Methods

Thin sections were cut parallel to the stretching lineation (X = L_s_ in the sketch shown in Figures and in Supplementary Figures) and perpendicular to the mylonitic foliation (Z parallel to the pole to the mylonitic foliation in the sketch shown in figures).

### Electron probe micro-analyser (EPMA) and X-ray compositional map elaboration

EPMA analysis was performed on carbon-coated thin sections using a JEOL JXA-8200 electron microprobe at the Department of Earth Sciences of the University of Milano (Italy). Backscattered electron images (BSE) were acquired using an accelerating voltage of 15 keV, a beam current of 5 nA, and a working distance of 11 mm. Point analyses and X-ray compositional maps were acquired using wavelength-dispersive spectrometers. Point analyses were acquired first, before the X-ray compositional maps on the same area. Analytical conditions of point analysis were a 15 keV accelerating voltage, a 5 nA beam current and a beam ø of ~ 1 μm. Nine oxide compositions were measured, using the following standards: grossular (SiO_2_/Al_2_O_3_/CaO), fayalite (FeO), forsterite (MgO), K-feldspar (K_2_O), omphacite (Na_2_O), ilmenite (TiO_2_), and rhodochrosite (MnO). Analytical conditions for Xray map acquisition were a 15 keV accelerating voltage, a 100 nA specimen current, and 50 ms dwell times. Nine elements (Si, Ti, Al, Fe, Mn, Mg, Na, Ca, and K) were measured at the specific wavelength in two passes. X-ray maps were processed using XMapTools^34^ and intensity X-ray maps were standardized to concentration maps of oxide weight percentage using spot analyses as the internal standard.

### Thermodynamic modelling

The Gibbs free energy minimization algorithm Theriak–Domino^59^ was used to compute the isochemical equilibrium phase diagrams, mineral isopleths, and diagrams of the H_2_O content in solids and modal amount of the hydrous mineral phases. The thermodynamic database of^60^, based on^61^, was used. All Gibbs free energy minimizations were computed with an excess in pure H_2_O fluid. Local bulk compositions were obtained using standardized X-ray maps, following the procedure described in^35^, removing the Fe content of hematite, as in^60^. Fe^3+^ was ignored because of its subtle content or absence in the studied mineral phases (Supplementary Table 2). Ca and Mn were removed from the input composition because of their minor content (Supplementary Table 3). Plots of vol% of the hydrous mineral phases and wt% of H_2_O in solids were computed along a prograde P–T path valid for the Northern Apennines, based on data from this study and from^23^ (Supplementary Fig. 5).

## Supplementary Information

Supplementary Information.

## Data Availability

All data generated or analysed during this study are available within this published article (and its Supplementary Information files).
