# Supplementary material for: A likely geological record of deep tremor and slow slip events from a subducted continental broken formation
Source: Sci Rep. 2022 Mar 16;12:4506. doi: 10.1038/s41598-022-08489-2 (PMC8927582; doi:10.1038/s41598-022-08489-2)
Supplement: Supplementary file 1 — Supplementary Information. [file 41598_2022_8489_MOESM1_ESM.docx]

# Supplementary information

**A likely geological record of deep tremor and slow slip events from a subducted continental broken formation**

Francesco Giuntoli ^1*^ & Giulio Viola ^1^

^1^ Department of Biological, Geological and Environmental Sciences, Università degli Studi di Bologna, Bologna, Italy.

* Corresponding author.

E-mail address: [francesco.giuntoli@unibo.it](mailto:francesco.giuntoli@unibo.it) (F. Giuntoli)

## Geological setting of the Giglio Island

The Giglio Island (Tuscany, Italy) is mostly composed of a 5 Ma old monzogranitic intrusion^1^. Tectonic slices of metamorphic rocks belonging to the Ligurian and Tuscan Metamorphic Units (of oceanic and continental affinity, respectively) crop out at the Franco Promontory, on the western side of the island^2,3^ (Supplementary Fig. 1). The contact between intrusive and metamorphic rocks is defined by a top-to-the W high-angle normal fault.

As reported in the main text, metasedimentary sequences from the Tuscan Metamorphic Units comprise the Middle-Low Triassic metasedimentary clastic rocks of the Verrucano Formation^4^ in tectonic contact with Upper Triassic limestone breccia (Calcare Cavernoso Formation), metalimestone and slate^2,3^ (Supplementary Fig. 1). The Verrucano Formation, the target of our study, is a well-known rock formation that crops out in the Northern Apennines on an area more than 200 km long (N-S) and 60 km wide (W-E), with a maximum reported thickness of 700 m at Monte Argentario^4,5^ (Supplementary Fig. 1). Pressure-temperature (P-T) conditions similar to those presented in this study were estimated for much of the Verrucano Formation, and range between 0.8-1.4 GPa and 350-450 °C (summary in Fig. 1 of ^6^ and references therein). Age data for the high-pressure metamorphism range from ~25 Ma for Verrucano metasediments at the Monte Argentario^7^ to 21-20 Ma for Tuscan Metamorphic Units on the Elba Island^6,8^.

Several metamorphic and deformation stages were recognised at the Giglio Island^2,3,9^, with the earliest being a relic blueschist facies foliation preserved within a retrograde greenschist facies foliation. This greenschist facies foliation is subhorizontal and is structurally associated with a stretching lineation trending E-NE/W-SW, non- cylindrical folds with axis parallel to the stretching lineation and top-to-the-E S-C structures and other consistent kinematic indicators. These structures were interpreted to reflect syn-orogenic extension, and the associated exhumation of the blueschist facies rocks during the local retrograde greenschist facies evolution^3,9^. As shown by us, however, in the study area the Verrucano is still predominantly associated with blueschist facies conditions with only static or local overprinting by greenschist facies fabrics (see below). Later, high-angle normal faulting accommodated further upper crustal and post-orogenic extension and facilitated pluton emplacement^3^.

## Petrographic and meso-microstructural analysis

Metaconglomerate, metaquartzarenite and metapelite alternate in the field as beds and bands with thickness ranging from a few metres to several tens of metres (Fig. 1a,b). The metaconglomerate and metaquartzarenite are commonly boudinaged within the weaker metapelite, with quartz and carpholite fibres growing parallel to the stretching lineation in the boudin necks (Fig. 1c,d and Fig. 2g,h). The three lithotypes contain a well-developed mylonitic foliation equilibrated at blueschist facies conditions (see next section), which parallels the lithological boundaries, leading to a composite fabric where the metamorphic foliation overprints and locally transposes the sedimentary bedding (Figs. 2 and 3; Supplementary Fig.2).

The metaconglomerate is generally massive and is composed of mostly rounded quartz clasts, from white to pink in colour, in a matrix of quartz, carpholite and muscovite (Fig. 2c,d and Supplementary Fig. 2a,b). The diameter of the clasts varies from few millimetres to some centimetres. The grain-supported primary sedimentary texture is still visible. The metaquartzarenite displays a characteristic white colour and a grain-supported sedimentary texture (Supplementary Fig. 2c,d). It envelops the metaconglomerate or forms bands in the metapelite (Fig. 1a,b and Supplementary Fig. 2). In both lithotypes, the mylonitic foliation wraps around the clasts and produces asymmetric pressure shadows containing quartz, carpholite and muscovite (Fig. 2c-f and Supplementary Fig. 2).

The metapelite is schistous with a shiny aspect on the foliation plane (Figs. 1e,f and 3 and Supplementary Fig. 2c,d). A composite foliation is seen in thin section, with predominant muscovite-rich bands and subordinate quartz and carpholite-rich bands (Fig. 3h-i). Muscovite grains appear dark due to graphite and hematite inclusions, some microns in size (Figs. 3d and 4c and Supplementary Fig. 3 and 4a). In all three lithotypes, a stretching lineation is present on the foliation and is marked by iso-oriented quartz, carpholite and muscovite grains (Figs. 2, 3, 5 and Supplementary Fig. 2c). Accessory rutile, hematite and tourmaline occur.

Two samples were analysed in detail acquiring and X-ray compositional maps: a dilational hydroshear vein (Sample A) and a metapelite (Sample B). Two generation of carpholite are distinguishable in sample A on the basis of microstructural analyses in combination with compositional X-ray mapping (Fig. 7a-c). The first generation is marked by a lower X_Mg_ carpholite (0.54-0.46; Supplementary Table 2) and is dissected by fractures perpendicular to its stretching direction. The second generation is characterised by a higher X_Mg_ (0.6-0.54) carpholite and grows along those fractures. This microstructure corroborates the proposed growth mechanism of the dilational hydroshear veins by incremental cracking and sealing.

Two generations of muscovite are also present in both samples we describe. The first generation is phengitic (Si apfu up to 3.35; High Si in Supplementary Table 2) and forms cores aligned along the blueschist facies foliation and relic grains in fold hinges (sample A; Fig. 7d and Supplementary Fig. 3d). The second generation is characterised by a lower Si content (Si apfu 3.2-3) and surrounds the previous cores (Sample A) or crystallizes along grain boundaries in association with chlorite (sample B; Fig. 7h and Supplementary Fig. 3h) and represents the retrograde greenschist facies overprint.

Also chlorite grows statically on the blueschist facies foliation, preferentially along the carpholite and muscovite-rich bands and in aggregates a few millimeters in size (Fig. 7e,f and Supplementary Fig. 6). Locally, the blueschist facies foliation is deformed by upright open to close folds with axis trending W-E, which contain discrete fractures in the hinge area filled with chlorite (Fig. 3f-i and Supplementary Figs. 6 and 7a) and by top-to-the E S-C-C’ structures with C and C’ planes decorated by chlorite (Supplementary Fig. 7b; see also^3^). Finally, two younger sets of conjugate low angle normal faults and high angle normal faults trending N-S and NNW-SSE deform all the previous structural elements (Supplementary Fig. 7c,d and Fig. 4 of ^3^). Low- and high angle normal fault planes are decorated by lenses of foliated and indurated silver-greenish to reddish gouge (Supplementary Fig. 7e).

## Thermodynamic modeling results

Local bulk compositions used for the isochemical phase diagram computations are available in Supplementary Table 3. The chosen areas used to extract the local bulk compositions correspond to the X‐ray maps of Fig. 7. In both samples, the modelled modal amounts (vol%) of mineral phases are comparable to the values observed in thin section for the same areas (Supplementary Table 3). The predicted assemblages, shown by the light-blue fields, match the observed parageneses and the estimated P-T conditions based on the computed isopleths (Fig. 8a,b). However, the lower part of the light-blue fields is characterised by the presence of chlorite. As described above chlorite overgrows the foliation marked by carpholite and muscovite or occurs in aggregates. Therefore, the fields Car, Ms, Pg, Chl, Rt and Qz are interpreted as marking the minimum P conditions for the development of the described structures, likely already marking the beginning of the retrograde path (mineral abbreviations as in^13^). The fields Car, Ms, Pg, Rt would indicate the stable parageneses during the development of the studied structures.

In Sample A, Si apfu (3.35-3.25) and X_Mg_ (0.7-0.5) isopleths of the first generation of phengitic muscovite intersect between 1.1-0.8 GPa and 350-300°C (High Si in Supplementary Table 2; Fig. 8a). These P-T conditions match with X_Mg_ isopleths of the first generation of carpholite (0.54-0.46). The second generation of carpholite with higher X_Mg_ (0.6-0.54) would indicate slightly higher temperature conditions than those constrained by the first one, suggesting prograde growth. The second generation of muscovite with lower Si apfu values (3.2-2.9) would suggest lower P conditions. We note that the measured X_Mg_ (0.5-0.3) value of this generation is lower compared to the values predicted by modeling (~0.7).

In Sample B, Si apfu (3.35-3.2) and X_Mg_ (0.7-0.55) isopleths of the first generation of muscovite intersect between 1.1-0.8 GPa and 350-300°C (High Si in Supplementary Table 2), similarly to sample A (Fig. 8b). These conditions match with X_Mg_ isopleths of carpholite (0.58-0.5). As for the previous sample, the second generation of muscovite display lower Si apfu (3.2-3) and would indicate lower P. The measured X_Mg_ (0.55-0.45) of this generation is lower compared to the values predicted by modeling (~0.75).

In summary, the P-T conditions computed for the development of both dilational hydroshear veins and foliation are between 1.1 GPa and 350° C and 0.8 GPa and 300° C (red dashed ellipses in Fig. 8a,b). P estimates are regarded as minimum values due to the presence of chlorite in the modeled parageneses, as in thin section chlorite is not observed stable with the other mineral phases (see previous section).

## Figures, tables and captions

**
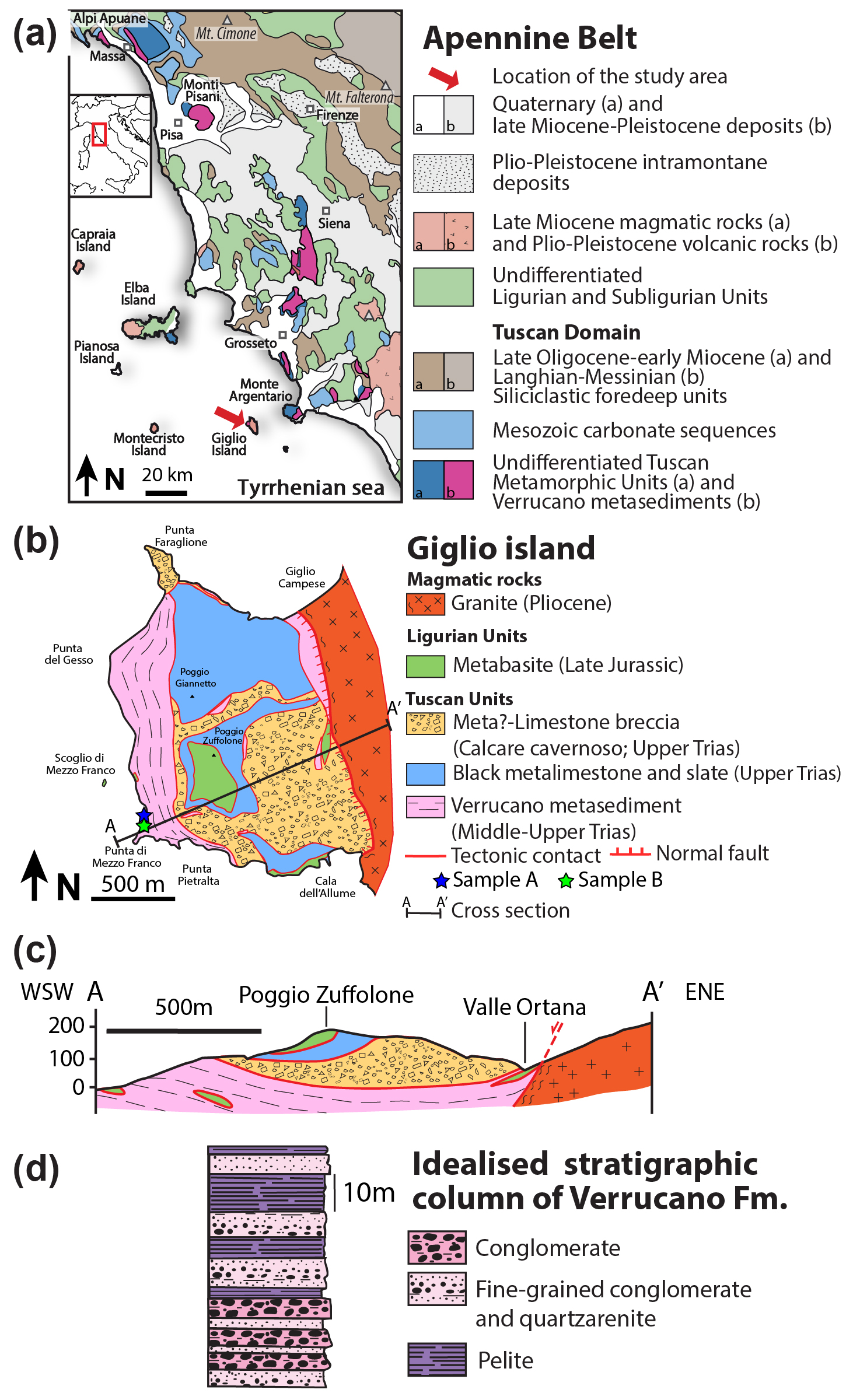
**

**Supplementary Figure 1. a**, Geological map of the Northern Apennines (based on^5^). **b**, Tectonic map of the Franco Promontory in the Giglio island (Tuscany, Italy; based on^2,3^). **c**, Geological cross section of the Franco Promontory (based on^2,3^). **d,** Idealised stratigraphic column of the Verrucano Formation, prior to Apennine metamorphism and deformation (based on^4^). Figure created with Adobe Illustrator CS6 (https://www.adobe.com/products/illustrator.html).


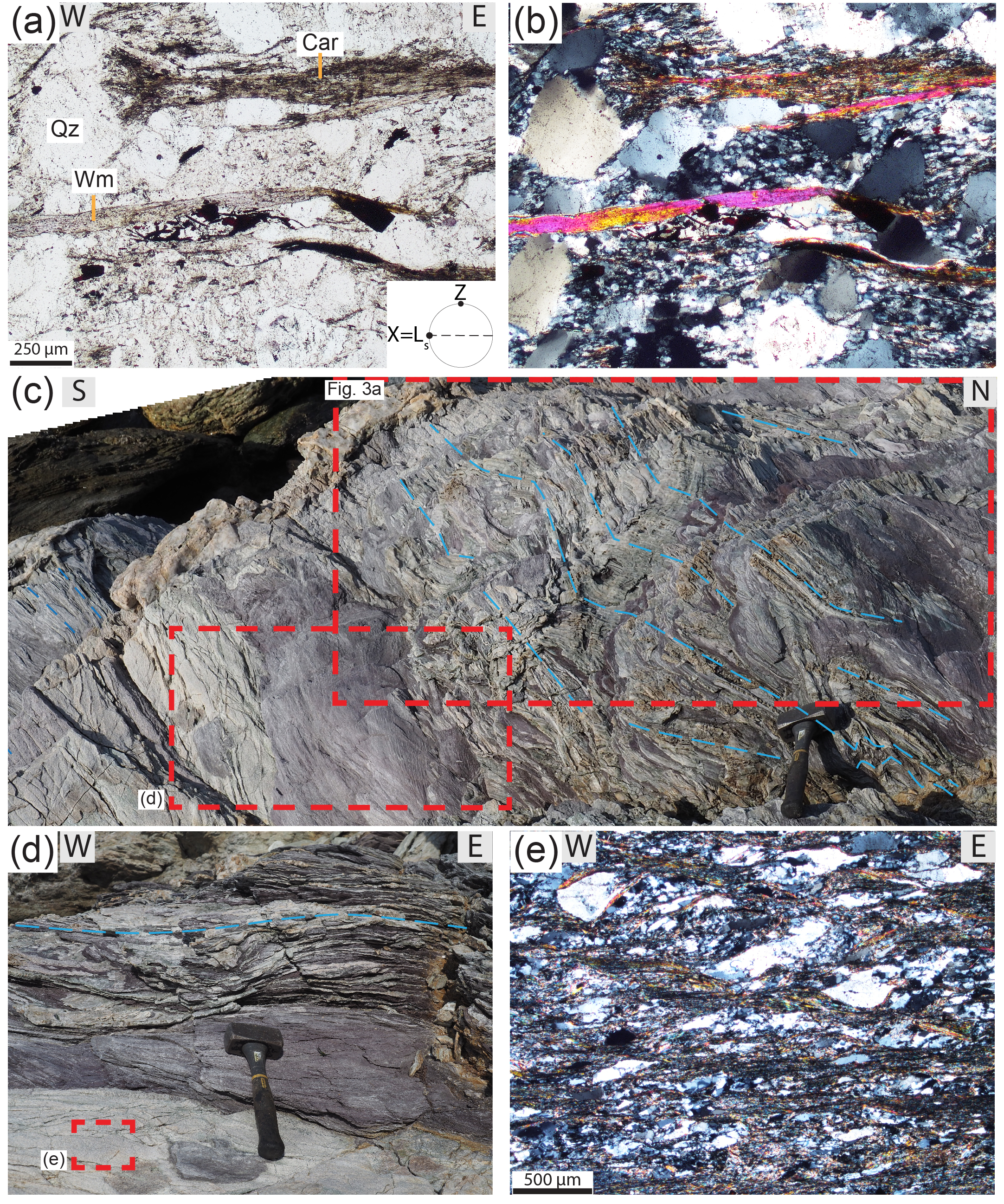


**Supplementary Figure 2.** **a, b,** Additional thin section photos of the metaconglomerate of Fig. 2d. Plane- and crossed-polarized light, respectively. **c,** Photograph of the outcrop of Fig. 3a taken parallel to the foliation with metapelite (violet) and quartz and carpholite dilational hydroshear veins in contact with a band of metaquartzarenite (white). Light blue dashed lines indicate the orientation of the fibres. **d**, View of the outcrop perpendicular to the foliation and parallel to the stretching lineation with a contact between metapelite at the top and metaquartzarenite at the bottom. The light blue dashed line indicates the trace of the blueschist facies foliation. **e**, Additional thin section photo of metaquartzarenite sheared by top-to-the-E blueschist facies extensional crenulation cleavage (sensu^14^). Crossed-polarized light.


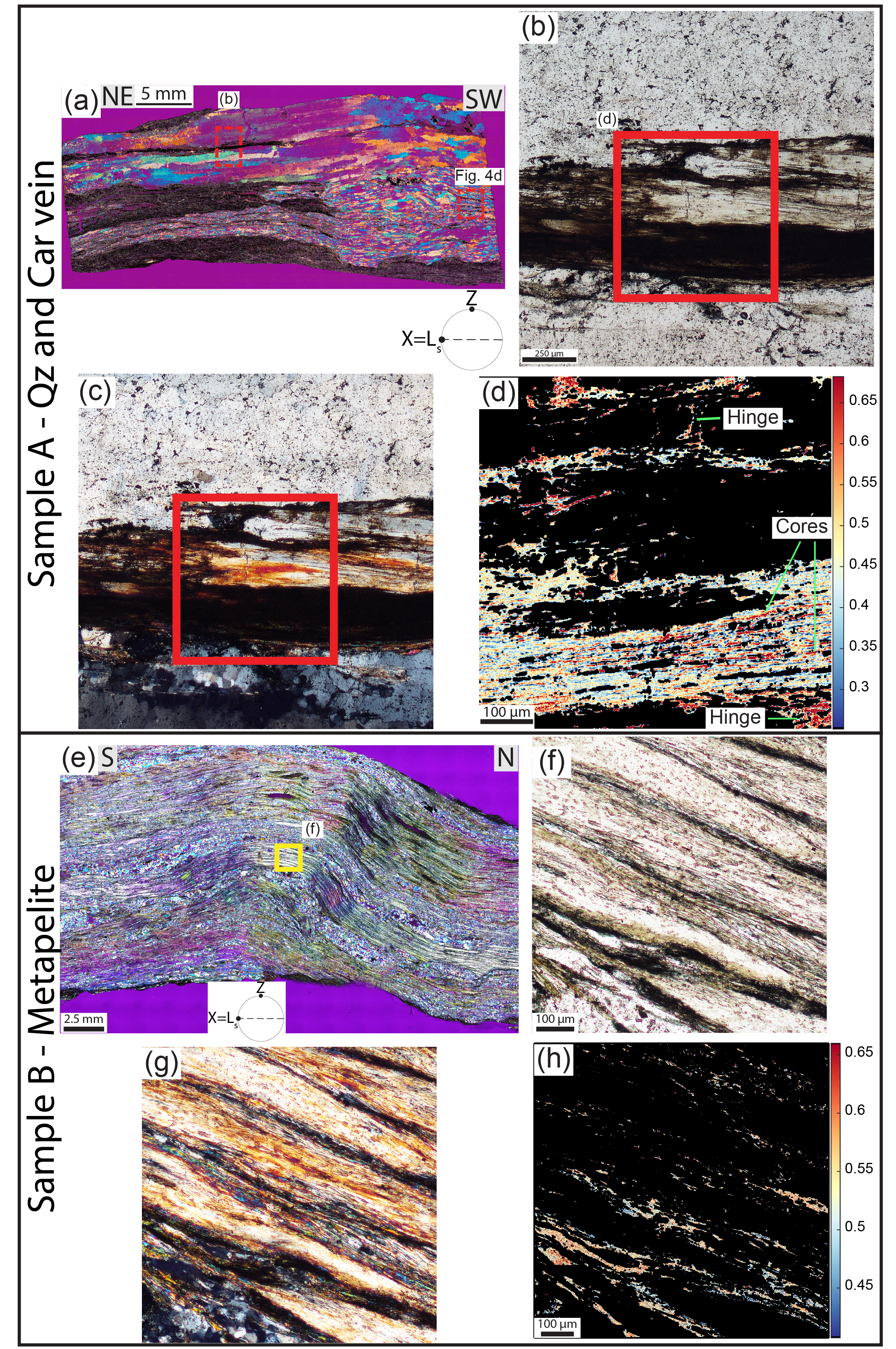


**Supplementary Figure 3.** Microstructural data of samples A (**a-d**) and B (**e-h**). **a**, Thin section optical scan highlighting the different grain size of the quartz fibres: a few centimetres in the upper part of the thin section, a few millimetres in the bottom-right portion and a few tens of microns in the bottom-left portion. Crossed-polarized light with gypsum plate inserted. **b**, **c**, Quartz and carpholite fibres in a dilational shear vein in contact with muscovite appearing dark due to graphite and hematite inclusions. Plane‐polarized light and crossed-polarized light, respectively. **d**, Standardised X-ray map of the X_Mg_ in muscovite highlights two muscovite generations: the first with higher values located in the core of the grains and in fold hinges and the second with lower values at grain boundaries. Compare with Fig. 7a-d. **e**, Foliation marked by quartz-rich bands and muscovite and carpholite-rich bands. Thin section optical scan, crossed-polarized light with inserted gypsum plate. **f**, **g**, Foliation marked by carpholite muscovite and quartz, with local retrogression marked by chlorite at grain boundaries. Plane‐polarized light and crossed-polarized light, respectively. **h**, Standardised X-ray map of X_Mg_ in muscovite. Compare with Fig. 7e-h.


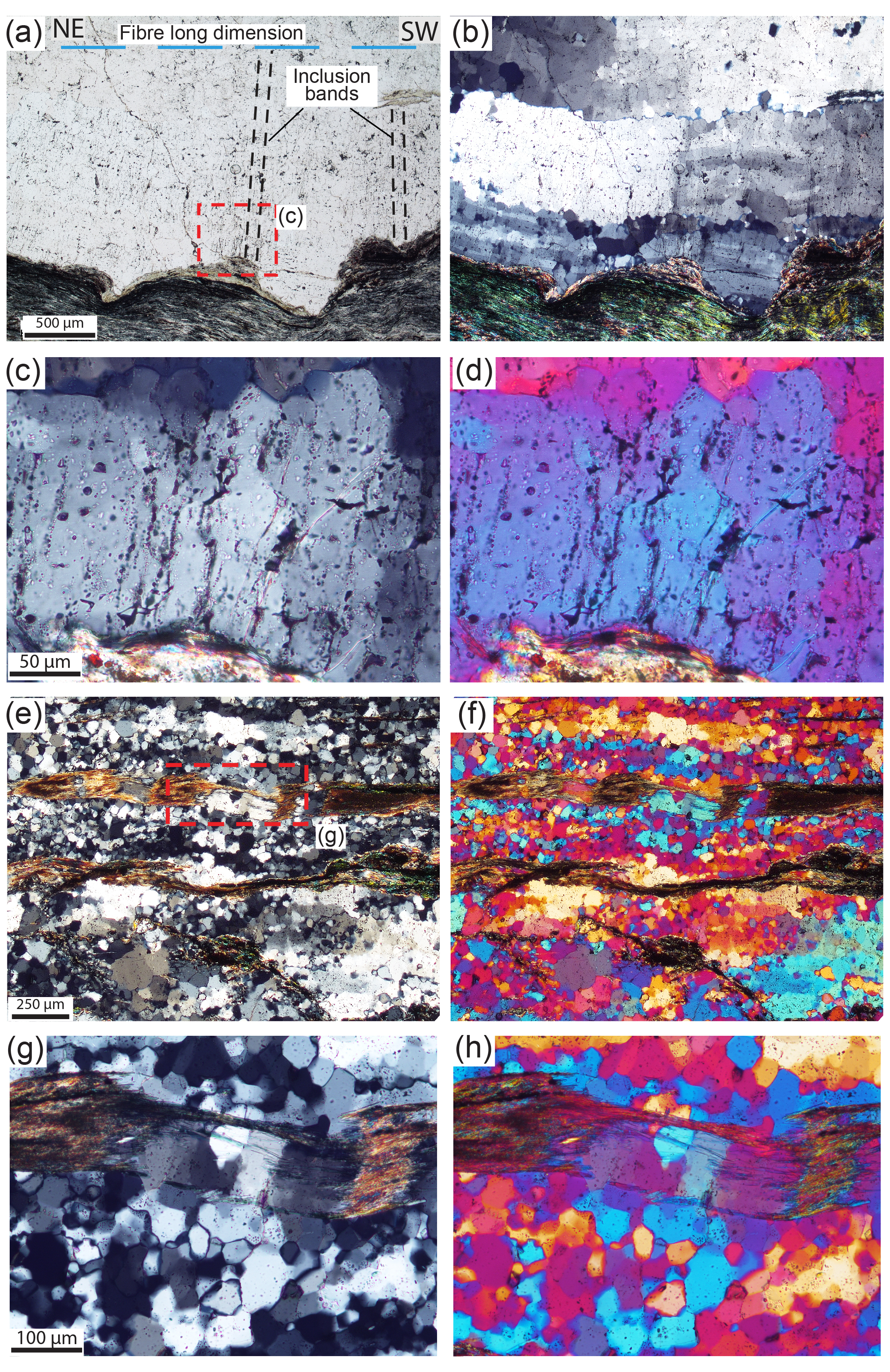


**Supplementary Figure 4. a-d**, Additional thin section photos of Fig. 4b,c. Inclusion bands (black dashed lines) in quartz-rich band. Plane- (a), crossed-polarized light (b,c) and crossed-polarized light with gypsum plate inserted (d). **e-h**, Additional thin section photos of Fig. 4d,e. Stretched and partly boudinaged carpholite fibres in dilational hydroshear vein. Crossed-polarized light (e,g) and crossed-polarized light with gypsum plate inserted (f,h).


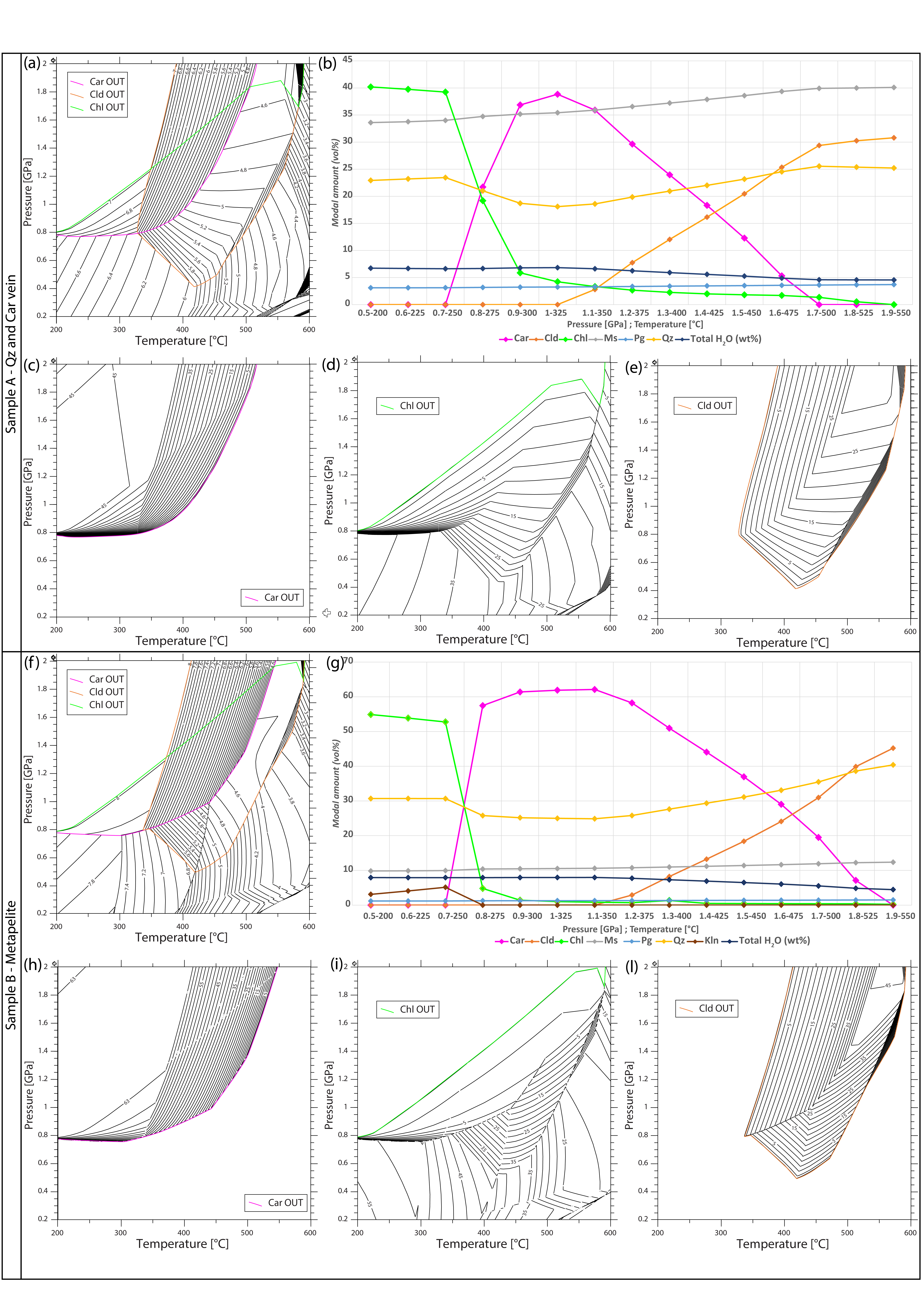


**Supplementary Figure 5.** Thermodynamic modelling of H_2_O content in solids (wt%) and modal amount of the hydrous mineral phases (vol%). **a**-**e**, Sample A. **a**, Wt% of H_2_O in solids. Carpholite, chloritoid and chlorite-out reactions are marked. **b**, Plot of the vol% of the hydrous mineral phases and wt% of H_2_O in solids (Total H_2_O) along the prograde P-T path of the Northern Apennines from 0.5 GPa and 200°C to 1.9 GPa and 550°C (based on data from this study and from^6^). **c-e**, Modal amount of carpholite, chlorite, and chloritoid, respectively (vol%). Note the correspondence between the decrease of H_2_O wt% in solids and the decrease of carpholite vol% (see text for explanation). **f-l**, Sample B. **f**, Wt% of H_2_O in solids. **g**, Plot of the modal amount of the hydrous mineral phases and wt% of H_2_O in solids. **h-l**, Modal amount of carpholite, chlorite, and chloritoid, respectively.

**
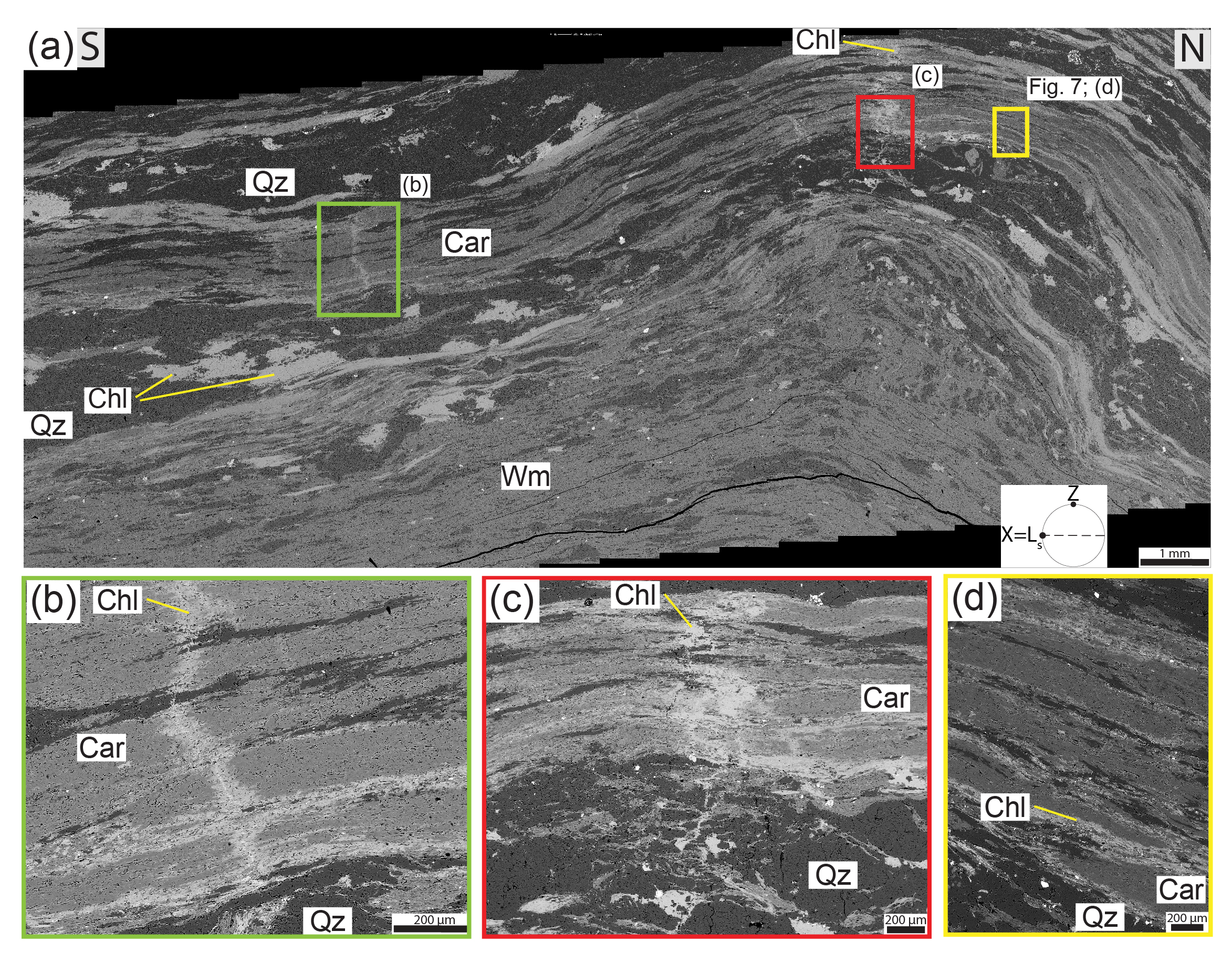
**

**Supplementary Figure 6.** Microstructures of metapelite sample B. **a**, BSE images of folded quartz-rich- and white mica and carpholite-rich bands. Chlorite grows along fractures in the fold hinge and limb (**b**, **c**) and overgrows the foliation-marking carpholite and muscovite at grain boundaries (**d**). Compare with Figs. 3f-i and Supplementary Fig. 3e-h.


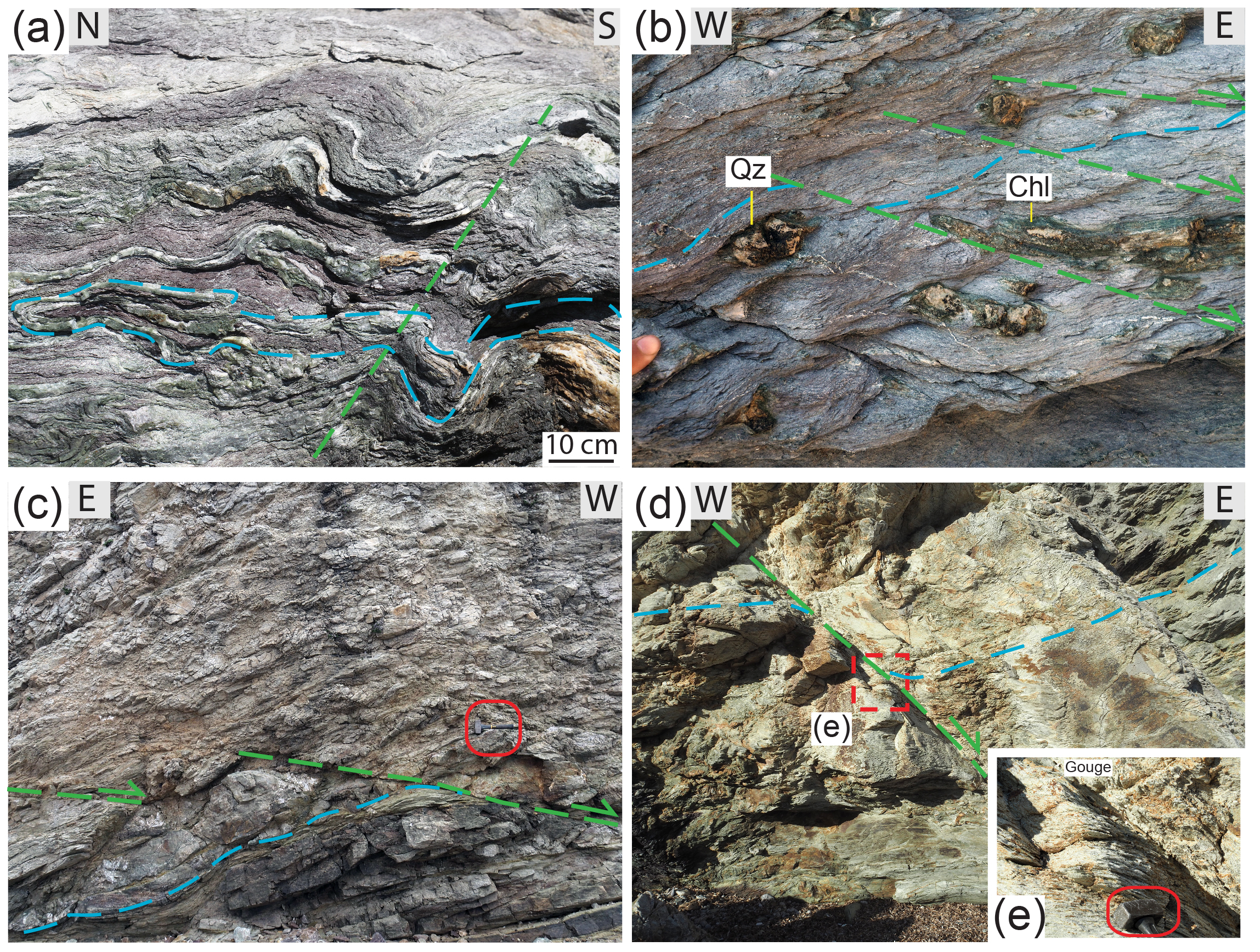


**Supplementary Figure 7.** Greenschist facies overprint. **a**, Intersection pattern between blueschist facies non-cylindrical fold in hydroshear veins (light blue dashed lines) and greenschist facies upright folds (the green dashed line marks the trace of the axial plane). **b***,* Metapelite with quartz clasts and greenschist facies C’ planes (green dashed lines) decorated by chlorite and indicating a top-to-the E sense of shear deforming the blueschist facies foliation (trace of foliation marked by light blue dashed lines). **c**, Discrete low angle extensional shear bands in metaquartzarenite; red circle: hammer for scale. **d**, High angle normal fault in metapelite. **e**, Detail of foliated and indurated silver-greenish to reddish gouge; red circle: hammer for scale.

| **Sample Name** | **GPS coordinates (Lat, Long)** |
| --- | --- |
| Sample A: Qz and Car vein | 42.354594N 10.867138E |
| Sample B: Metapelite | 42.354327N 10.866948E |

**Supplementary Table 1**. Sample names and GPS coordinates.

|  | **Ms** | | | | | | |  | **Car** | | | | | | **Chl** | |
| --- | --- | --- | --- | --- | --- | --- | --- | --- | --- | --- | --- | --- | --- | --- | --- | --- |
| **Sample** | **A** | | | | **B** | | | | **A** | | | | **B** | | **A** | **B** |
| **Wt%** | **High Si** | | **Low Si** | | **High Si** | | **Low Si** | | **High Mg** | | **Low Mg** | |  |  |  |  |
|  | Av Comp | σ | Av Comp | σ | Av Comp | σ | Av Comp | σ | Av Comp | σ | Av Comp | σ | Av Comp | σ | Spot Analysis | Spot Analysis* |
| SiO_2_ | 50.24 | 1.36 | 44.64 | 1.37 | 49.08 | 1.11 | 46.02 | 1.72 | 39.12 | 0.96 | 39.42 | 1.01 | 39.26 | 1.92 | 29.12 | 31.08 |
| TiO_2_ | 0.13 | 0.05 | 0.17 | 0.06 | 0.18 | 0.09 | 0.15 | 0.07 | 0.40 | 0.40 | 0.26 | 0.28 | 0.12 | 0.12 | 0.07 | 0.05 |
| Al_2_O_3_ | 31.96 | 1.01 | 34.23 | 1.06 | 32.53 | 0.91 | 32.59 | 1.41 | 31.64 | 0.85 | 31.24 | 0.88 | 31.71 | 1.49 | 21.02 | 21.18 |
| FeO | 1.94 | 0.98 | 5.95 | 2.19 | 1.92 | 0.29 | 2.99 | 1.14 | 10.25 | 0.77 | 11.67 | 0.72 | 10.54 | 0.71 | 21.87 | 23.40 |
| MnO | 0.04 | 0.03 | 0.06 | 0.04 | 0.03 | 0.02 | 0.04 | 0.02 | 0.13 | 0.05 | 0.13 | 0.05 | 0.20 | 0.11 | 0.49 | 1.09 |
| MgO | 1.59 | 0.33 | 2.59 | 0.73 | 1.40 | 0.19 | 1.71 | 0.52 | 7.59 | 0.49 | 6.84 | 0.51 | 6.92 | 0.42 | 16.54 | 11.34 |
| CaO | 0.09 | 0.04 | 0.20 | 0.05 | 0.18 | 0.21 | 0.12 | 0.03 | 0.04 | 0.03 | 0.04 | 0.03 | 0.09 | 0.10 | 0.14 | 0.19 |
| Na_2_O | 1.00 | 0.58 | 0.62 | 0.41 | 0.58 | 0.178 | 1.18 | 0.53 | 0.02 | 0.02 | 0.03 | 0.02 | 0.01 | 0.01 | 0.08 | 0.05 |
| K_2_O | 9.81 | 0.36 | 7.64 | 0.83 | 9.69 | 0.364 | 8.66 | 0.59 | 0.03 | 0.05 | 0.07 | 0.12 | 0.02 | 0.01 | 0.01 | 1.72 |
| Total | 96.79 | – | 96.09 | – | 95.59 | – | 93.47 | – | 89.22 | – | 89.71 | – | 88.86 | – | 89.34 | 90.10 |
| **Formulae based on** | **on 11 O** | | | | | | |  | **on 8 O** | | | | | | **on 14 O** | |
| Si | 3.28 | 0.05 | 2.98 | 0.07 | 3.24 | 0.04 | 3.13 | 0.08 | 2.02 | 0.04 | 2.04 | 0.04 | 2.04 | 0.06 | 2.88 | 3.07 |
| Ti | 0.01 | 0.00 | 0.01 | 0.00 | 0.01 | 0.00 | 0.01 | 0.00 | 0.02 | 0.02 | 0.01 | 0.01 | 0.00 | 0.00 | 0.01 | 0.00 |
| Al | 2.46 | 0.09 | 2.70 | 0.15 | 2.53 | 0.07 | 2.61 | 0.13 | 1.93 | 0.04 | 1.91 | 0.05 | 1.94 | 0.08 | 2.48 | 2.52 |
| Fe^2+^ | 0.11 | 0.07 | 0.33 | 0.12 | 0.11 | 0.03 | 0.17 | 0.07 | 0.44 | 0.03 | 0.51 | 0.03 | 0.46 | 0.03 | 1.63 | 1.67 |
| Fe^3+^ | 0.00 | 0.00 | 0.00 | 0.00 | 0.00 | 0.00 | 0.00 | 0.00 | 0.00 | 0.00 | 0.00 | 0.00 | 0.00 | 0.00 | 0.20 | 0.30 |
| Mn | 0.00 | 0.00 | 0.00 | 0.00 | 0.00 | 0.00 | 0.00 | 0.00 | 0.01 | 0.00 | 0.01 | 0.00 | 0.01 | 0.01 | 0.04 | 0.09 |
| Mg | 0.15 | 0.03 | 0.26 | 0.08 | 0.14 | 0.03 | 0.17 | 0.07 | 0.59 | 0.04 | 0.53 | 0.04 | 0.54 | 0.03 | 2.44 | 1.67 |
| Ca | 0.01 | 0.00 | 0.01 | 0.00 | 0.01 | 0.00 | 0.01 | 0.00 | 0.00 | 0.00 | 0.00 | 0.00 | 0.01 | 0.01 | 0.01 | 0.02 |
| Na | 0.13 | 0.07 | 0.08 | 0.05 | 0.07 | 0.02 | 0.16 | 0.07 | 0.00 | 0.00 | 0.00 | 0.00 | 0.00 | 0.00 | 0.03 | 0.02 |
| K | 0.82 | 0.03 | 0.65 | 0.07 | 0.82 | 0.03 | 0.75 | 0.05 | 0.00 | 0.00 | 0.01 | 0.01 | 0.00 | 0.00 | 0.00 | 0.43 |
| ∑ cations | 6.96 | – | 7.03 | – | 6.93 | – | 7.01 | – | 5.00 | – | 5.00 | – | 4.99 | – | 9.73 | 9.80 |
| X_Mg_ | 0.59 | 0.17 | 0.44 | 0.13 | 0.56 | 0.05 | 0.51 | 0.16 | 0.57 | 0.02 | 0.51 | 0.02 | 0.54 | 0.02 | 0.60 | 0.50 |
|  |  |  |  |  |  |  |  |  |  |  |  |  |  |  |  | *Chloritised muscovite |

**Supplementary Table 2**. Representative spot and average composition analyses (wt%) of the mineral phases. Fe^3+^ and Fe^2+^ contents of carpholite following^15^. Fe^3+^ and Fe^2+^ contents of muscovite and chlorite calculated assuming full tetrahedral site occupancy.

| **Sample A - Quartz and carpholite vein** | | |
| --- | --- | --- |
| **Assemblage Theriak @ 330°C-1GPa** | Vol% | Car 38.1%, Ms 35.5%, Qz 18.3%, Chl 4.7%, Pg 3.2%, Rt 0.17% |
| **Estimated in the X-ray compositional map** | Vol% | Wm 47%, Car 34%, Qz 19% |
| **Estimated in the entire thin section** | Vol% | Qz 45%, Wm 30%, Car 15%, Chl 5%. Remaining 5%: Hem, Cb, Rt, Gr |
|  |  |  |
| **Local bulk composition (No Hem)** | Wt% | SiO_2_ (53.1583)Al_2_O_3_(26.0149)FeO(6.7817)MnO(0.0721)MgO(3.7164)CaO(0.1039)Na_2_O(0.2926)TiO_2_(0.23931)K_2_O(4.0172) |
|  |  |  |
| **Input Theriak: No Ca, Mn and excess H_2_O** | | Si(0.8846)Al(0.5103)Fe(0.0944)Mg(0.0922)Na(0.0094)Ti(0.0031)K(0.0853)H(1)O(?) |
| **Sample B - Metapelite** | | |
| **Assemblage Theriak @ 330°C-1GPa** | Vol% | Car 61.7%, Qz 25%, Ms 10.56%, Pg 1.27%, Chl1.17%, Rt 0.29% |
| **Estimated in the X-ray compositional map** | Vol% | Car 63%, Qz 22%, Wm 12%, Chl 3% |
| **Estimated in the entire thin section** | Vol% | Wm 35%, Car 25%, Qz 25%, Chl 10%. Remaining 5%: Hem, Rt, Gr |
|  |  |  |
| **Local bulk composition** | Wt% | SiO_2_ (53.3588)Al_2_O_3_(24.2734)FeO(7.2716)MnO(0.1380)MgO(4.6657)CaO(0.1151)Na_2_O(0.1087)TiO_2_(0.4054)K_2_O(1.1709) |
|  |  |  |
| **Input Theriak: No Ca, Mn and excess H_2_O** | | Si(0.8880)Al(0.4761)Fe(0.1012)Mg(0.1158)Na(0.0035)Ti(0.0052)K(0.0249)H(1)O(?) |

**Supplementary Table 3**. Comparison of modelled and estimated mineral assemblages (in volume %) at the estimated P-T conditions of mylonitic foliation and dilational hydroshear veins development. Input bulk compositions are given.

## References supplementary information

1. Westerman, D. S., Innocenti, F., Tonarini, S. & Ferrara, G. The Pliocene intrusions of the Island of Giglio. *Mem. della Soc. Geol. Ital.* **49**, 345–363 (1993).

2. Lazzarotto, A., Mazzanti, R. & Mazzoncini, F. Geologia del promontorio Argentario (Grosseto) e del promontorio del Franco (Isola del Giglio-Grosseto). *Boll. Soc. Geol. Ital* **83**, l-l24 (1964).

3. Rossetti, F. *et al.* Syn-versus post-orogenic extension: the case study of Giglio Island (Northern Tyrrhenian Sea, Italy). *Tectonophysics* **304**, 71–93 (1999).

4. Azzaro, E. *et al.* Geology and Petrography of the Verrucano and Paleozoic Formations of Southern Tuscany and Northern Latium (Italy). in *The Continental Permian in Central, West, and South Europe* (ed. Falke, H.) 181–195 (Springer Netherlands, 1976). doi:10.1007/978-94-010-1461-8_15

5. Conti, P., Cornamusini, G. & Carmignani, L. An outline of the geology of the Northern Apennines (Italy), with geological map at 1: 250,000 scale. *Ital. J. Geosci.* **139**, 149–194 (2020).

6. Bianco, C. *et al.* The lawsonite-glaucophane blueschists of Elba Island (Italy). *Lithos* **348**, 105198 (2019).

7. Brunet, C., Monié, P., Jolivet, L. & Cadet, J.-P. Migration of compression and extension in the Tyrrhenian Sea, insights from 40 Ar/39 Ar ages on micas along a transect from Corsica to Tuscany. *Tectonophysics* **321**, 127–155 (2000).

8. Ryan, E. *et al.* Syn-Orogenic Exhumation of High-P Units by Upward Extrusion in an Accretionary Wedge: Insights From the Eastern Elba Nappe Stack (Northern Apennines, Italy). *Tectonics* **40**, e2020TC006348 (2021).

9. Jolivet, L. *et al.* Midcrustal shear zones in postorogenic extension: example from the northern Tyrrhenian Sea. *J. Geophys. Res. Solid Earth* **103**, 12123–12160 (1998).

10. Cox, S. F. & Etheridge, M. A. Crack-seal fibre growth mechanisms and their significance in the development of oriented layer silicate microstructures. *Tectonophysics* **92**, 147–170 (1983).

11. Fagereng, Å., Remitti, F. & Sibson, R. H. Incrementally developed slickenfibers — Geological record of repeating low stress-drop seismic events? *Tectonophysics* **510**, 381–386 (2011).

12. Ujiie, K. *et al.* An Explanation of Episodic Tremor and Slow Slip Constrained by Crack-Seal Veins and Viscous Shear in Subduction Mélange. *Geophys. Res. Lett.* **45**, 5371–5379 (2018).

13. Whitney, D. L. & Evans, B. W. Abbreviations for names of rock-forming minerals. *Am. Mineral.* **95**, 185–187 (2010).

14. Platt, J. P. & Vissers, R. L. M. Extensional structures in anisotropic rocks. *J. Struct. Geol.* **2**, 397–410 (1980).

15. Droop, G. T. R. A general equation for estimating Fe3+ concentrations in ferromagnesian silicates and oxides from microprobe analyses, using stoichiometric criteria. *Mineral. Mag.* **51**, 431–435 (1987).
